# Electrochemical multi-analyte point-of-care perspiration sensors using on-chip three-dimensional graphene electrodes

**DOI:** 10.1007/s00216-020-02939-4

**Published:** 2020-09-28

**Authors:** Meike Bauer, Lukas Wunderlich, Florian Weinzierl, Yongjiu Lei, Axel Duerkop, Husam N. Alshareef, Antje J. Baeumner

**Affiliations:** 1 Institute of Analytical Chemistry, Chemo- and Biosensors, University of Regensburg, 93040 Regensburg, Germany; 2 Physical Science and Engineering Division, King Abdullah University of Science and Technology (KAUST), Thuwal, 23955-6900 Kingdom of Saudi Arabia; 3 Department of Biological and Environmental Engineering, Cornell University, Ithaca, NY 14853 USA

**Keywords:** Laser-induced graphene (LIG), Point-of-care (POC), Electrochemical biosensor, Sweat sensor, Health-monitoring platform

## Abstract

Multi-analyte sensing using exclusively laser-induced graphene (LIG)-based planar electrode systems was developed for sweat analysis. LIG provides 3D structures of graphene, can be manufactured easier than any other carbon electrode also on large scale, and in form of electrodes: hence, it is predestinated for affordable, wearable point-of-care sensors. Here, it is demonstrated that LIG facilitates all three electrochemical sensing strategies (voltammetry, potentiometry, impedance) in a multi-analyte system for sweat analysis. A potentiometric potassium-ion-selective electrode in combination with an electrodeposited Ag/AgCl reference electrode (RE) enabled the detection of potassium ions in the entire physiologically relevant range (1 to 500 mM) with a fast response time, unaffected by the presence of main interfering ions and sweat-collecting materials. A kidney-shaped interdigitated LIG electrode enabled the determination of the overall electrolyte concentration by electrochemical impedance spectroscopy at a fixed frequency. Enzyme-based strategies with amperometric detection share a common RE and were realized with Prussian blue as electron mediator and biocompatible chitosan for enzyme immobilization and protection of the electrode. Using glucose and lactate oxidases, lower limits of detection of 13.7 ± 0.5 μM for glucose and 28 ± 3 μM for lactate were obtained, respectively. The sensor showed a good performance at different pH, with sweat-collecting tissues, on a model skin system and furthermore in synthetic sweat as well as in artificial tear fluid. Response time for each analytical cycle totals 75 s, and hence allows a quasi-continuous and simultaneous monitoring of all analytes. This multi-analyte all-LIG system is therefore a practical, versatile, and most simple strategy for point-of-care applications and has the potential to outcompete standard screen-printed electrodes.

**Electronic supplementary material:**

The online version of this article (10.1007/s00216-020-02939-4) contains supplementary material, which is available to authorized users.

**Figure:**
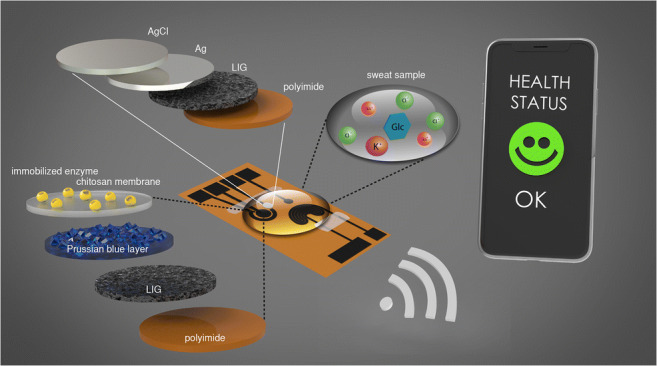
Graphical abstract

## Introduction

The pursuit of developing bio- and chemosensors has long been driven by the realization that these sensors have a powerful potential to address the analytical challenges of onsite, rapid, accurate, simple, and inexpensive detection. Through miniaturization, advancements in biorecognition elements, in coating chemistries, and signal amplification new sensor designs indeed keep proving exactly this potential. A quite current trend in onsite diagnostics seeks to develop wearable sensors not only for clinical diagnostics but also for monitoring of fitness or health state [1]. Here, the miniaturization of electronic components and development of new materials are equally key to advance wearable sensing technology further, as can be evidenced by products ranging from smart watches and wristbands which monitor heartbeat or body temperature through adhesive stickers and screen-printed tattoos to smart textiles and contact lenses which are capable of collecting more information than physical vital signs [2].

In the year 1953, Paul di Sant’ Agnese et al. published the first article on the detection of cystic fibrosis, a genetic disorder, in context of increased salt concentration in sweat and saliva [3]. Thus, standard test procedures for sodium chloride content in sweat for the immediate detection of the pernicious disease were developed [4]. Sweat also contains many different electrolytes, and other relevant biomarkers like organic acids, metal ions, amino acids, carbohydrates, and vitamins which can be used for drawing conclusions on an individual’s health status [5, 6]. Therefore, besides the sensitivity of the respective detection method for biomarkers in lower concentration ranges, the selectivity of the receptor of the sensor is very important for reliable measurements.

Easy accessibility of sweat at any time with non-invasive collecting methods qualifies sweat as a perfect bio matrix for point-of-care (POC) diagnostics and long-term health monitoring. In general, many factors like environment, activity level, hormones, sympathetic nervous system, and also the individual diet induce perspiration [7]. Eccrine sweat is generated easily over the entire body during physical activities or without exercising in warmer surroundings to regulate the body temperature [8]. For subjects at rest, the sweat rate may be too low for continuous monitoring. However, perspiration can be stimulated simply with heat or by iontophoretic techniques using low electrical currents in combination with agonists like pilocarpine [9]. The analysis of sweat is predestined to be realized in a wearable device for continuous monitoring over a long-term period. As such, reliable glucose level diagnosis by sweat analysis would improve millions of diabetics’ daily live routine in a significantly more comfortable way. Studies reveal promising results that the glucose concentration in sweat correlates with blood glucose level and contains no glucose from the environment, albeit studies indicate that blood and interstitial analysis are more reliable [7, 10].

A light-weighted and in the best case not noticeable wearable sweat sensor is the favorable way to perform real-time monitoring of body conditions for medical home-care applications or during physical activity. Hydration level, the related electrolyte content, and concentrations of biomolecules give a good overview of the current state of the user’s physiological conditions. Potassium ions in general are required for correct nerve transmission and an oversupply as well as a lack thereof can cause several effects up to an abnormal heart rhythm and finally death [11]. An increased electrolyte content in sweat during workout is a direct indication for dehydration [12]. Lactate is an important biomarker for providing information on the oxygen supply in tissue and the entire anaerobic metabolism in muscles. Its concentration in sweat correlates directly with the concentration in blood [13]. If anomalies in these biomarker concentrations are detected in a timely manner, a fast-acting electrolyte or drug intake can prevent malfunctions of the body like muscle cramps or much more threatening consequences occurring due to nutrient deficits [14].

Sweat analysis of such analytes can easily be performed through electrochemical detection. Furthermore, inexpensive mass production possibilities, miniaturization, integration into fluidic systems, and with flexible circuit boards make electrochemical detection a preferred technology for sweat analysis. In fact, carbon-based electrodes dominate the electrochemical point-of-care market [15–20] and advances and better understanding of graphene suggests that it is a highly favorable transducer material.

In search of economic and straight-forward synthesis routes for graphene, Lin et al. reported the new concept of laser-induced graphene (LIG) also termed laser-scribed graphene (LSG) by others [21–23]. It was made from commercial polymers such as polyimide (PI) derivatives in 2014 by exposing those to a computer-controlled CO_2_ infrared laser which generates 3D graphene layers under ambient conditions [24]. This economic and reagent-free one-step synthesis of porous graphene on a polymer substrate opened incredible new applications for bioanalytical demands [21–23, 25–28]. It was thus studied here as a material for sweat-based sensing. Of special interest was to demonstrate that an all-LIG sensor can be generated for multi-analyte detection, employing the three important electrochemical detection strategies of voltammetry, potentiometry, and conductometry. Through LIG surface modifications, enzyme-based biosensors for glucose and lactate, an ion-selective chemosensor for potassium ions, and a simple conductometric electrolyte setup were developed. The multi-analyte performance was evaluated to prove that it functioned well within the physiological ranges of all analytes in sweat and was tested on skin and in artificial sweat samples.

## Materials and methods

### Chemicals

All solid and liquid chemicals were of at least analytical grade and purchased from Sigma-Aldrich (Sigma-Aldrich Chemie GmbH, Taufkirchen, Germany), Fluka (Fluka Chemie GmbH, Buchs, Switzerland), VWR (VWR International GmbH, Darmstadt, Germany), Merck (Merck KGaA, Darmstadt, Germany), or Roth (Carl Roth GmbH + Co. KG, Karlsruhe, Germany). Chitosan from crab shells (practical grade) was purchased from Sigma-Aldrich. All reagents were used without further purification. Millipore water was used for preparation of all aqueous solutions.

### Enzymes

Lactate oxidase (LOx) type II (from *Aerococcus viridans*, 36.0 U mg^−1^ powder) was purchased from Hölzel Diagnostika (Hoelzel Diagnostika Handels GmbH, Cologne, Germany). Glucose oxidase (GOx) type VII (from *aspergillus niger*, 168.8 kU mg^−1^ powder) was purchased from Sigma-Aldrich.

### Other materials

Polyimide film with a thickness of 125 μm was purchased from CMC Klebetechnik (CMC Klebetechnik GmbH, Frankenthal, Germany). As sweat-collecting pads, the following materials were used: Kimtech Science labor tissues (Kimberly-Clark GmbH, Koblenz, Germany), Whatman® 595 filter paper (Schleicher & Schuell, Dassel, Germany), gauze (EAZ GmbH, Boeblingen, Germany). Chicken leg was purchased from local supermarkets. Different nail polishes, kitchen towels, and artificial tear fluid were purchased from local drug stores.

### Equipment and accessories

The following equipment with suitable software and accessories were used: Keithley 175 autoranging multimeter (Keithley Instruments Inc., Cleveland, Ohio, USA), CHI 650 A potentiostat (CH Instruments Inc., Austin, Texas, USA), portable bipotentiostats/galvanostats μStat400 (Metrohm DropSens, Filderstadt, Germany), PalmSens4, and EmStat Blue (PalmSens BV, GA Houten, Netherlands). The handheld plating device was purchased from Conrad Electronic (Conrad Electronic SE, Hirschau, Germany). Commercially available Ag/AgCl reference electrodes (Bioanalytical Systems Inc., West Lafayette, IN, USA) and a Pt wire (Goodfellow GmbH, Hamburg, Germany) were used. A Dino Lite digital USB microscope with suitable software DinoCapture 2.0 was used to capture magnified images of the modified LIG electrodes (Dunwell Tech, Inc., Torrance, CA, USA). For all laser-scribing processes, a laser-engraving device VLS 2.30 equipped with a 30 W CO_2_ laser (λ = 10.6 μm) from Universal Laser Systems (Universal Laser Systems Inc., Scottsdale, Arizona, USA) was used. For imaging the morphology and structure of the laser-induced graphene, a scanning electron microscope (SEM) LEO 1530 from Zeiss (Carl Zeiss AG, Oberkochen, Germany) was used. Raman spectroscopy was performed with a DXR Raman microscope from Thermo Fisher (Thermo Fisher Scientific GmbH, Dreieich, Germany).

### Preparation of solutions and buffers

1X PBS (phosphate-buffered saline) solution with pH 7.4, 0.1 mol L^−1^ citrate buffers with pH 4 and 5, 0.1 mol L^−1^ phosphate buffers with pH 6 and 7, and a potassium-free phosphate buffer (0.1 mol·L^−1^, pH 7.4) for the multi-analyte sensor were prepared with and without 1 mmol L^−1^ glucose. All buffers were stored at 4 °C.

A potassium chloride stock solution with concentration of 1 mol L^−1^ was prepared in water and diluted to concentrations ranging between 1·10^−7^ and 0.5 mol L^−1^. For the interfering cation study, MgCl_2_, CaCl_2_, and NaCl stock solutions with a concentration of 0.1 mol L^−1^ were prepared in 10 mmol L^−1^ KCl solution and diluted with 10 mmol L^−1^ KCl solution. AgNO_3_ solution for silver deposition with mass concentration β = 400 mg mL^−1^ in water was prepared. For the K^+^-selective membrane, two precursor solutions were made: solution A: 270 mg polyvinylchloride (PVC) were dissolved in 2.7 mL of tetrahydrofuran (THF). Solution B: 520 μL dibutyl sebacate (DBS) as plasticizer was mixed with 50 μL of valinomycin solution (β = 80 mg mL^−1^ in methanol). Solution B was continuously stirred while solution A was added. The membrane solution contained 16 mg of valinomycin ionophore per gram PVC. A second PVC cocktail without valinomycin was prepared as described before as protection membrane for the reference electrode. These solutions were stored in a THF atmosphere at 4 °C and have to be stirred for at least 1 h at room temperature before use. Nafion solution was mixed with an excess of solid KCl and continuously stirred to obtain a saturated solution.

For Prussian blue deposition on the working electrode, two 10 mmol L^−1^ iron (III) salt solutions (precursor solutions) were prepared from FeCl_3_ and K_3_[Fe(CN)_6_]. Both were dissolved in a 0.1 mol L^−1^ HCl solution containing 0.1 mol L^−1^ KCl. Storage at 4 °C in the dark is recommended. A 0.1% weight chitosan solution was prepared by dissolving chitosan in 0.1 mol L^−1^ acetic acid. A 100 mmol L^−1^ hydrogen peroxide (HP) stock solution was freshly prepared before use. Dilution series of HP in buffer ranging between 1 μmol L^−1^ and 10 mmol L^−1^ were prepared. Synthetic sweat solution according to DIN 53160-2 was prepared by dissolving 5 g NaCl and 1 g urea in 1 L water and its pH was adjusted with 1% NH_4_OH solution to 6.5. For the biosensor assays, 100 mmol L^−1^ D-glucose and sodium L-lactate stock solutions were prepared and diluted with the respective buffers to a concentration range between 1 μmol L^−1^ and 10 mmol L^−1^. D-Glucose solutions were allowed to mutarotate overnight. Lactate solutions in concentrations ranging between 10 μmol L^−1^ and 10 mmol L^−1^ were additionally prepared in synthetic sweat and artificial tear fluid. Lyophilized enzymes were dissolved in PBS. The activity of the solution of glucose oxidases was 4 U μL^−1^. For lactate oxidase, an activity of 5 U μL^−1^ was adjusted.

### Laser-scribing process and general electrode treatment

The desired electrode structures were drawn in original size with the vector graphic software CorelDRAW 17 (Corel Corporation, Ottawa, Ontario, Canada). The CorelDRAW data are compatible with the software of the used laser-engraving device VLS 2.30 (Universal Laser Systems Inc., Scottsdale, Arizona, USA) and needs no further conversion. The electrodes were fabricated on a 125-μm-thick polyimide film. The scribing was performed by exposing the film to a 30 W CO_2_ laser with 1% laser power and 10% scribing speed (maximum speed is 0.127 m s^−1^). The image density parameter was set to level 7, representing 1000 laser pulses per inch (PPI) in x-direction and 2000 PPI in y-direction. Figure S1 in the Electronic Supplementary Material (ESM) shows the schematic procedure of the laser-scribing process. Further optimizations of the scribing process are explained in the ESM. The electrodes were cleaned with water and isopropyl alcohol and dried with compressed air to remove dust traces around the pattern. The strands were isolated with two layers of nail polish. Connection pads were protected with copper tape or silver paint.

### Characterization of electrode material

Morphology and graphene-like characteristics of the LIG were determined by SEM and Raman spectroscopy (see ESM, Fig. S23). Morphology was studied by SEM at 5.0 kV. The samples have been cut with a scissor and gold sputtered for 30 s (≈ 7 nm layer thickness) after placing them on specimen stubs. Raman spectra from 50 to 3500 cm^−1^ were collected on a Raman microscope with a 532-nm laser set to 8 mW power and a 50× objective with an estimated focal spot diameter of 0.7 μm. Sixteen scans were averaged per spot.

### Fabrication of the potentiometric sensor

A simple design with two circular electrodes was used. Eight microliters of the ISE membrane cocktail was deposited onto one electrode to coat the whole electrode area. The membrane was dried overnight at ambient conditions. For RE fabrication, 20 μL of AgNO_3_ solution (β = 400 mg∙mL^−1^) was dropped on the RE and equilibrated on the electrode for 5 min. A commercial handheld plating device with a voltage of 3 V and a current of 300 mA was used. The plating process was carried out for 1 min. The electrode was immersed into a saturated KCl solution and a 0.8 V (vs. Ag/AgCl) DC voltage was applied for 200 s to receive the silver chloride layer on top. The entire modification process is schematically shown in Fig. S9 (see ESM). For further information regarding the optimization process, see ESM.

### Design of the impedance sensor

An applicable design with interdigitated two-electrode structure was developed and optimized (see ESM). The sensor needed no further modifications.

### Amperometric biosensor with layered working electrode

**Fig. 1:**
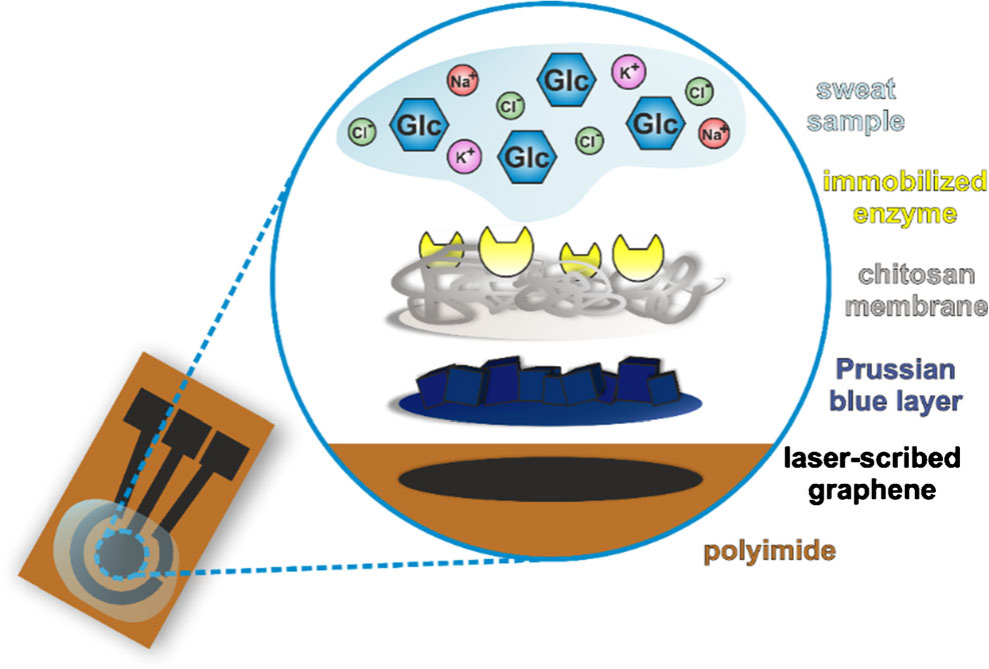
Scheme of the WE of the amperometric biosensor after all modification steps. The laser-induced graphene on the flexible PI substrate is the base for the chemical deposited PB layer. The chitosan membrane fixes the PB layer while providing the polymer network on which the enzyme is immobilized. The sweat sample or any other solution is applied on top

A three-electrode setup based on commercially available planar sensor systems was chosen. Two microliters of each iron salt precursor solution was applied exclusively onto the working electrode (WE) area and allowed to react there for 20 min under ambient conditions. The formed PB layer is stabilized for 2 h at 100 °C. The chitosan membrane was applied by drop-coating 2 μL of the chitosan solution on the PB-modified WE. The membrane was dried for 90 min under ambient conditions. Two microliters of the respective enzyme solution was applied to the dried chitosan membrane. For incubation, the electrodes were stored at least 2 h at ambient conditions, or overnight at 4 °C. A scheme of the entire buildup of the modified WE is shown in Fig. 1. Reference electrode (RE) and counter electrode (CE) were made from unmodified LIG. After cleaning with a few droplets of buffer solution, the sensors were ready to use.

### Multi-analyte design

**Fig. 2:**
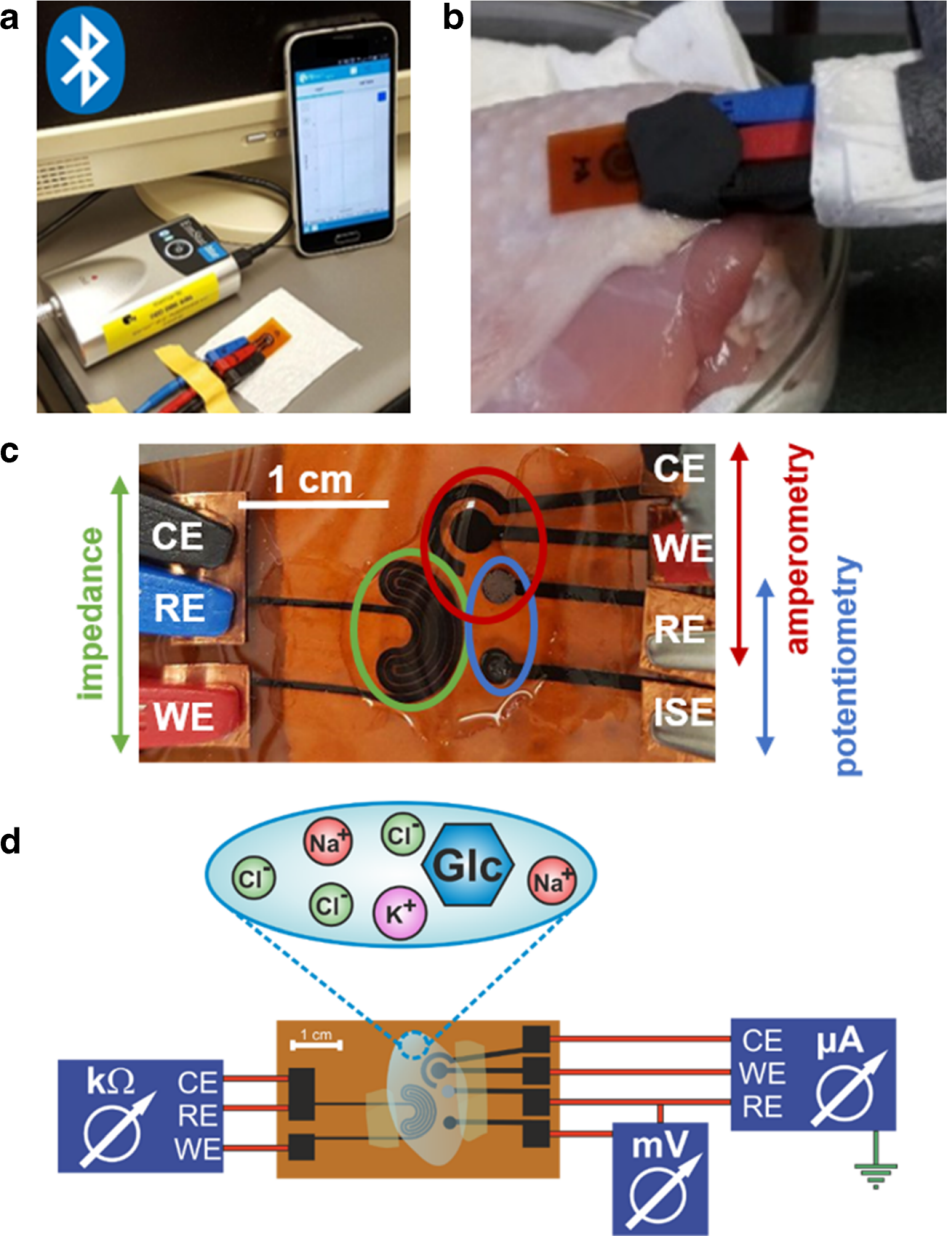
**a** Sensor connected to the EmStat Blue potentiostat via three crocodile clamps attached to the connection pads. For planar setup, a sample droplet of 30 μL which covers all three electrodes was added. There is a wireless connection of the potentiostat via Bluetooth® to the software application installed on a smartphone. **b** Experimental setup for detection of glucose and lactate with amperometric measurements on a chicken leg to simulate human skin and to provide a biological substrate. A piece of filter paper is used as simulated sweat collection pad. Modeling clay protects the contacts from moisture due to the shortened strands. The sensor is in a fixed position, whereas the chicken substrate with the applied sample can be moved up and down. **c** Combined LIG sensor. Connection pads are protected with adhesive copper tape against abrasion through the crocodile clamps. The strands are isolated by nail polish. 150 μL to 200 μL of sample solution is suitable to cover the active electrode area. **d** Schematic view of the combined LIG biosensor on polyimide substrate connected to two potentiostats and a multimeter. 150 μL to 200 μL of mixed samples containing all potential analytes is applied and can be measured quasi simultaneously after calibration of the single sensors

The three single-analyte sensor designs were combined so that the amperometric and potentiometric sensors share a circular RE with electrodeposited Ag/AgCl layer like shown in Fig. 2c.

### General procedures and instrumental settings

For potentiometric measurements, both electrodes were connected to a multimeter, 30 μL of sample solution was applied, and the potential was read off immediately. The sensor was carefully swabbed with tissue and the next solution was applied. The same procedure was performed with all other sample solutions. Sample application without using sweat collection pads is herein called droplet method. In case of using filter paper or gauze as sample collection pad, sample volume was doubled.

For impedance measurements, 100 μL of the respective ion solution was applied and the measurement was started at a fixed frequency of 1000 Hz with an AC amplitude of 10 mV and DC potential of 0 V, immediately. After the measurement was finished, the solution was carefully swabbed away with a paper towel and the respective following solution with increasing analyte concentration was applied.

The amperometric sensor was connected to a commercial software-controlled potentiostat. For the general procedure, a droplet of 30 μL of the respective sample solution was applied to the biosensor (with and without filter paper as sweat collection substrate) and the measurement was started immediately. Different experimental setups are shown in Fig. 2a and b. The working range of the sensors was determined by measuring a cyclic voltammogram (CV) of PBS between 0.85 V and − 0.95 V vs. LIG with a scan speed of 50 mV s^−1^. The CVs showed usually four characteristic peaks of the respective reduction and oxidation steps of the PB mediator layer. A potential in the decay of the first oxidation peak, usually between − 0.1 V and 0.1 V vs. LIG, was chosen for the amperometric determinations. If the Ag/AgCl RE was used, the CV settings were adjusted to a slightly higher potential. With the same experimental setup, the chronoamperometric measurements were performed with a run time of 60 s and the potential determined from the CV. To equilibrate the sensor, buffer was measured for at least five times. The measurement was started immediately after sample application without further incubation time. The droplet on the electrode was carefully absorbed with a paper towel and the next sample solution was applied.

For the multi-analyte sensor, 150 μL of sample solution containing different amounts of each analyte was applied to the multi-electrode system. For a quasi-simultaneous acquisition, the potential difference of the ISE was read out first after sample application. Then, the impedance was measured and as last step, the amperometric detection was done. In summary, one analytical cycle takes approximately 75 s. A schematic experimental setup is shown in Fig. 2c and d.

Some measurements were performed with filter paper or gauze as sweat collection pad and on a chicken leg with skin to simulate detection under more real conditions (see Fig. 2b for the experimental setup). Moreover, lactate quantification was performed in synthetic sweat solution according to DIN 53160-2 and in artificial tear fluid.

### Statistics and data evaluation

All calculations, especially the arithmetic mean values and standard deviation (SD), were calculated with Microsoft Excel 2016 (Microsoft Corporation, Redmond, Washington, USA). Usually, measurements were performed at least in triplicate (*n* = 3). Suspicious values were removed after failing the outlier Q-test. SD is represented by error bars in y-direction.

Linear and non-linear regression curves were accomplished with following Eqs. (1) and (2). Lower limit of detection (LOD) and lower limit of quantification (LOQ) for linear calibration curves were calculated with Eqs. (3) and (4). To calculate the LOD and LOQ from a sigmoidal fit curve, the Eqs. (5) and (6) were used.

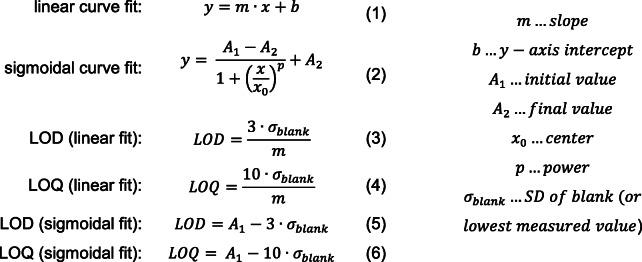



## Results and discussion

The development strategy of the multi-analyte all-LIG sensor is based on an initial study of each single sensing principle separately. Electrode layout, laser-scribing conditions, and all other depending parameters and procedures were optimized to serve all three sensing concepts well. The key was to demonstrate that an all-LIG concept is possible for all three electroanalytical detection strategies and that those can easily be combined for multi-analyte detection.

### Potentiometric sensor for potassium ion quantification

**Fig. 3:**
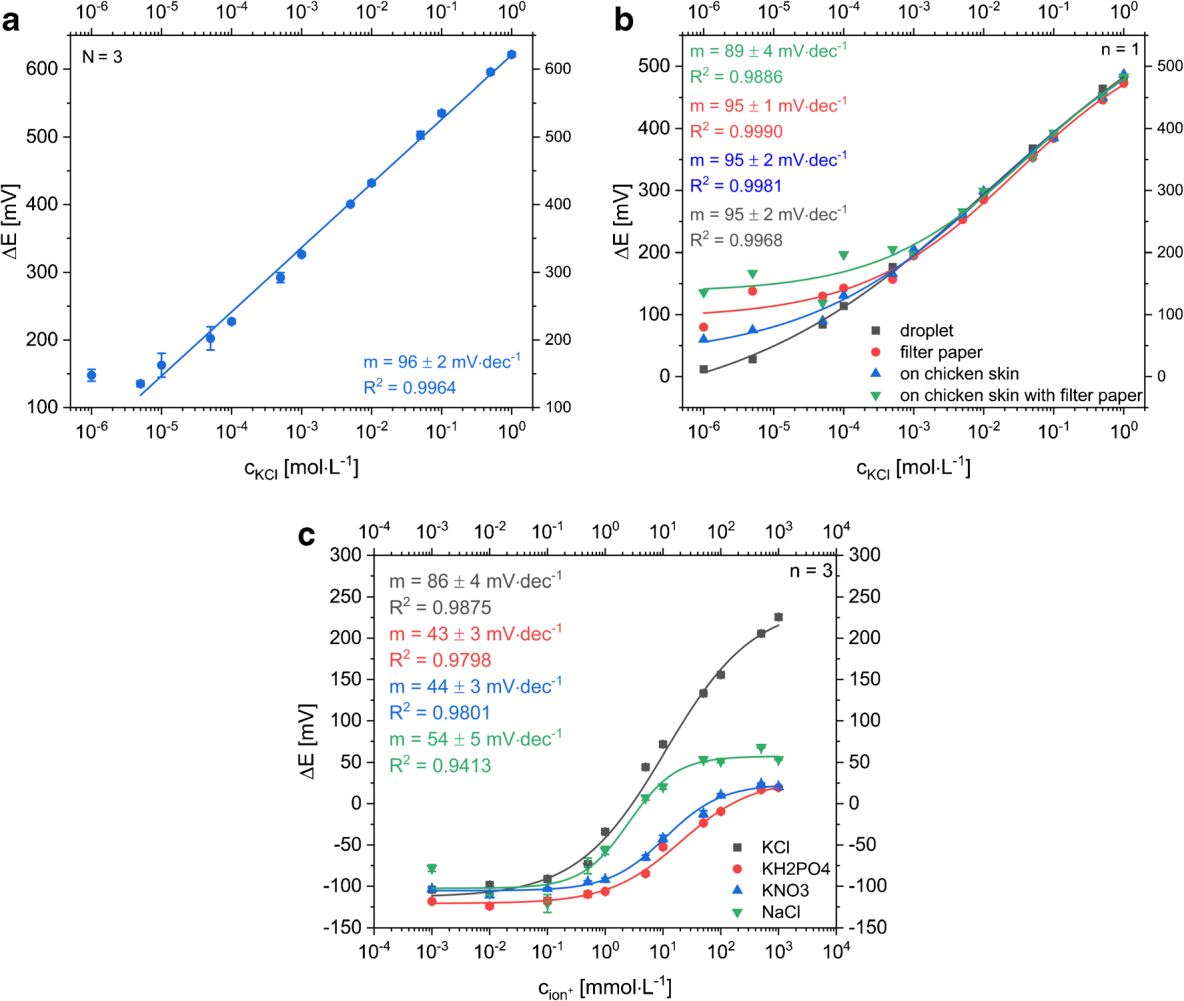
**a** Dose-response curve of a potentiometric, planar LIG-based sensor with Ag/AgCl RE measuring KCl concentrations ranging from 10^−6^ to 1 mol L^−1^ with the droplet method (N_sensor_ = 3). The linear range goes down to 1·10^−5^ mol L^−1^ KCl and the slope is 96 ± 2 mV·dec^−1^. The small standard deviation, represented as error bars, especially in the linear range, indicates a reproducible electrode fabrication procedure. **b** Dose-response curves of the potentiometric LIG sensor with Ag/AgCl RE. KCl samples were measured by application of droplets (gray boxes), using a filter paper as sweat collection pad (red circles), measuring sample droplets on chicken skin (blue triangle) and on chicken skin with filter paper (green triangle). **c** Dose-response curves of one sensor when different electrolytes containing K^+^ and Cl^−^ ions are measured to demonstrate the sensor’s sensitivity towards both species (gray boxes: KCl, red circles KH_2_PO_4_, blue triangles: KNO_3_ green triangles: NaCl)

The LIG-based sensor for potentiometric determination of potassium ions consists of an ion-selective electrode with valinomycin embedding PVC membrane and an electrodeposited Ag/AgCl reference electrode. The potential of the reference electrode fabricated via galvanic silver plating and subsequent electrochemical oxidation in saturated KCl solution was measured vs. a silver chloride–coated silver wire. A difference below 1 mV indicated a well-working production process. A planar design with circular electrodes was chosen considering a future application as sweat sensor worn on skin. Size, distance, and shape of the electrodes were optimized for best performance (see ESM, Figs. S3–S9) resulting in a well-working all-LIG sensor with relative errors below 4% (Fig. 3). Specifically, K^+^ detection worked within a concentration range of 1·10^−5^ mol L^−1^ to 1 mol L^−1^ KCl when covering the electrodes with a single droplet of sample solution. Using filter paper as simulated sweat collection pad, the lower linear range limit increased to 1 mmol L^−1^ KCl indicating an expected direct negative impact on the ion diffusion profile to the sensor surface. As the median range of interest for K^+^ analysis in sweat is fluctuating around 5 mmol L^−1^ with peak values up to 38 mmol L^−1^ [5, 29, 30], the dynamic range of the sensor is an excellent fit. Furthermore, due to valinomycin, a good selectivity towards K^+^ ions was demonstrated compared with the main interfering cations Na^+^, Mg^2+^, and Ca^2+^ as shown in Fig. S5 (see ESM).

As can be seen in Fig. 3a and b, the slopes of the dose-response curves are around 95 mV·dec^−1^ in case of using the electrodeposited Ag/AgCl RE. This indicates that our pseudo Ag/AgCl reference electrode contributes to the signal and responds to the chloride ions of the KCl standards accordingly. This was demonstrated by simply performing the same experiments with KNO_3_, NaCl, and KH_2_PO_4_ (Fig. 3c), whereas the other electrolytes showed just half of the slope in contrast to the dose-response curve for KCl.

**Fig. 4:**
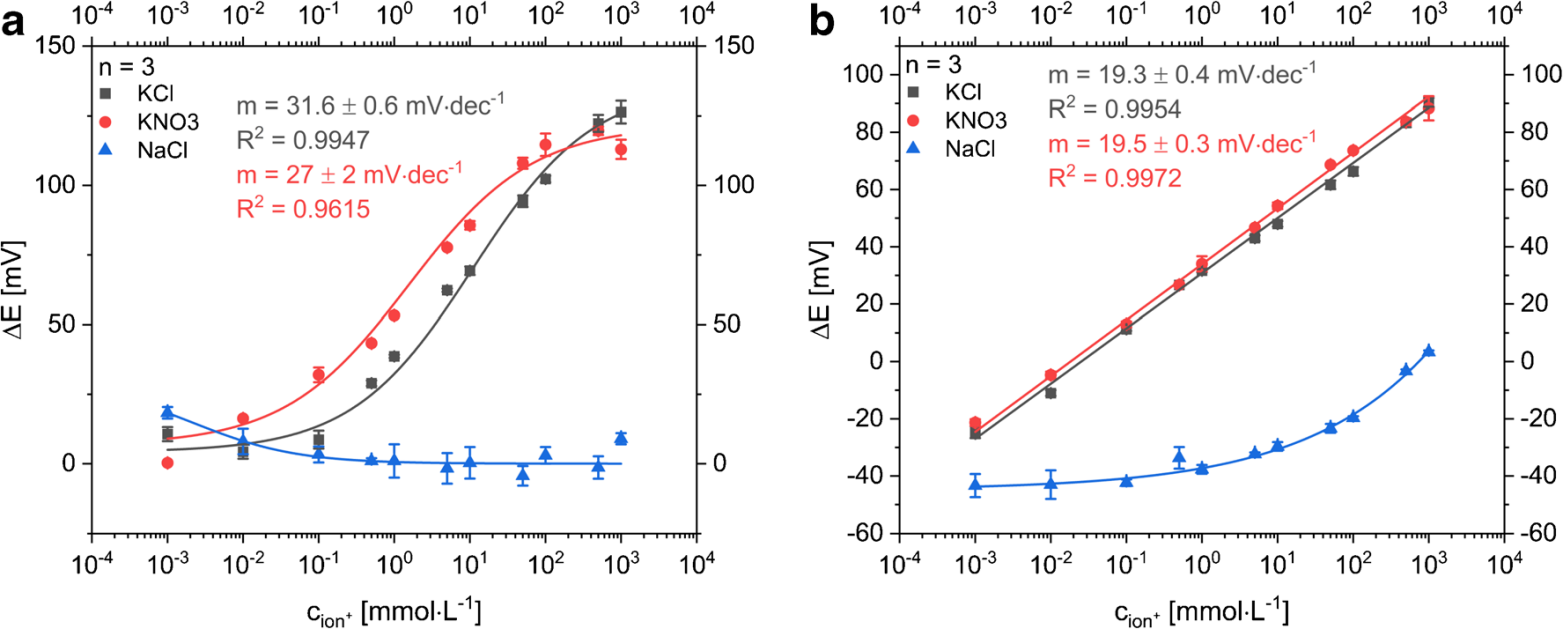
Dose-response curves of all-LIG potentiometric sensors with an ISE versus different RE **a** Ag/AgCl pseudo-RE was protected by a Nafion/KCl/PVC layer and **b** an unmodified LIG was used as RE material to minimize the influence of chloride ions (gray boxes: KCl, red circles KNO_3_, blue triangles: NaCl; *n* = 3)

In theory, a 118 mV slope (i.e., 2× Nernstian slope of 59 mV) would be expected for KCl additions. However, the pre-deposition of AgCl on the pseudo reference electrode likely prevents that. For actual K^+^ ion quantification in sweat, two strategies can be used. Either a simple LIG electrode can be applied as reference material or the pseudo-RE will be covered with a Nafion/KCl/PVC membrane (Fig. 4). The additional membrane keeps chloride concentration at the RE constant and prevents Cl^−^ ions from the sample to interfere. Besides the reduced sensitivity caused by the increased resistance, both strategies show a suitable way to minimize the influence of chloride ions within the relevant range. Moreover, we expect that the unprotected Ag/AgCl electrode in combination with another RE works as simple chloride ion sensor.

It should be pointed out that the overall fabrication of the potentiometric sensors is reliable even on lab-scale as shown by minimal differences in slopes and low standard deviations (Figs. 3, 4, and 8). However, it can also be seen that each change in sensor setup creates additional resistance layers which is reflected in the varying slopes observed in these data sets, i.e., sensors without Nafion layer follow a Nernstian behavior, as those with additional PVC and Nafion layers, or when mechanically disturbed by filter paper or gauzes show an overall decrease in slope.

### Impedance sensor for electrolyte quantification

**Fig. 5:**
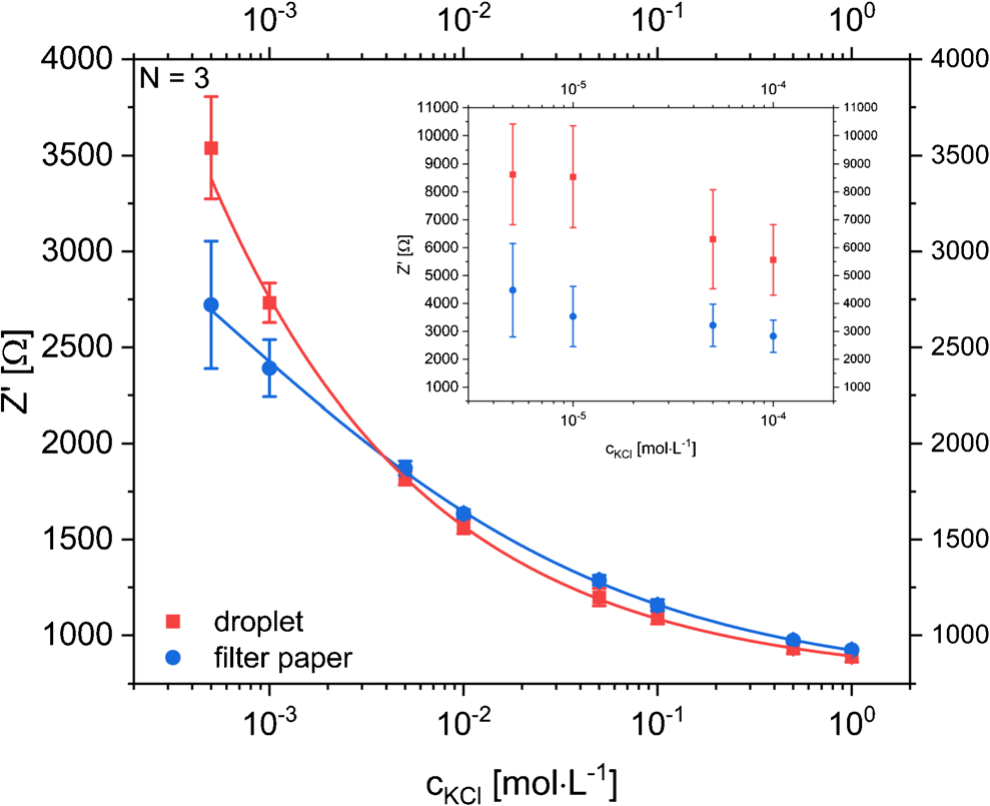
Dose-response curve of impedance measurements of KCl solutions with concentrations ranging between 5·10^−4^ and 1 mol L^−1^ in a planar all-LIG setup (N_sensor_ = 3) with the droplet method (blue circles) and Whatman® 595 filter paper (red boxes) to simulate a sweat collection pad. The inset shows a zoomed-in section for the concentration range below the relevant range. Instrumental settings: fixed frequency of 1000 Hz with an AC amplitude of 10 mV and DC potential of 0 V

EIS was used to quantify the overall electrolyte content in sweat. Electrolyte concentration in sweat is an important indicator for the hydration level of the body. Therefore, an interdigitated LIG electrode structure was designed and optimized (see ESM, Fig. S10–S15). The impedance measurement with the kidney-shaped sensor was performed at constant frequency of 1 kHz with a fixed amplitude of 10 mV to enable a fast response of the sensor. Application of KCl solutions to the sensor with and without filter paper as sweat collection pad showed small standard deviations and similar curve shapes in the physiological-relevant electrolyte concentration range of 1 mmol L^−1^ to 1 mol L^−1^ [5] (see Fig. 5). Ion concentrations in sweat range from lower millimolar concentrations up to peak values over 0.5 mol L^−1^ which is well within the reliable detection range of this sensor [5, 12].

### Amperometric glucose and lactate biosensors

**Fig. 6:**
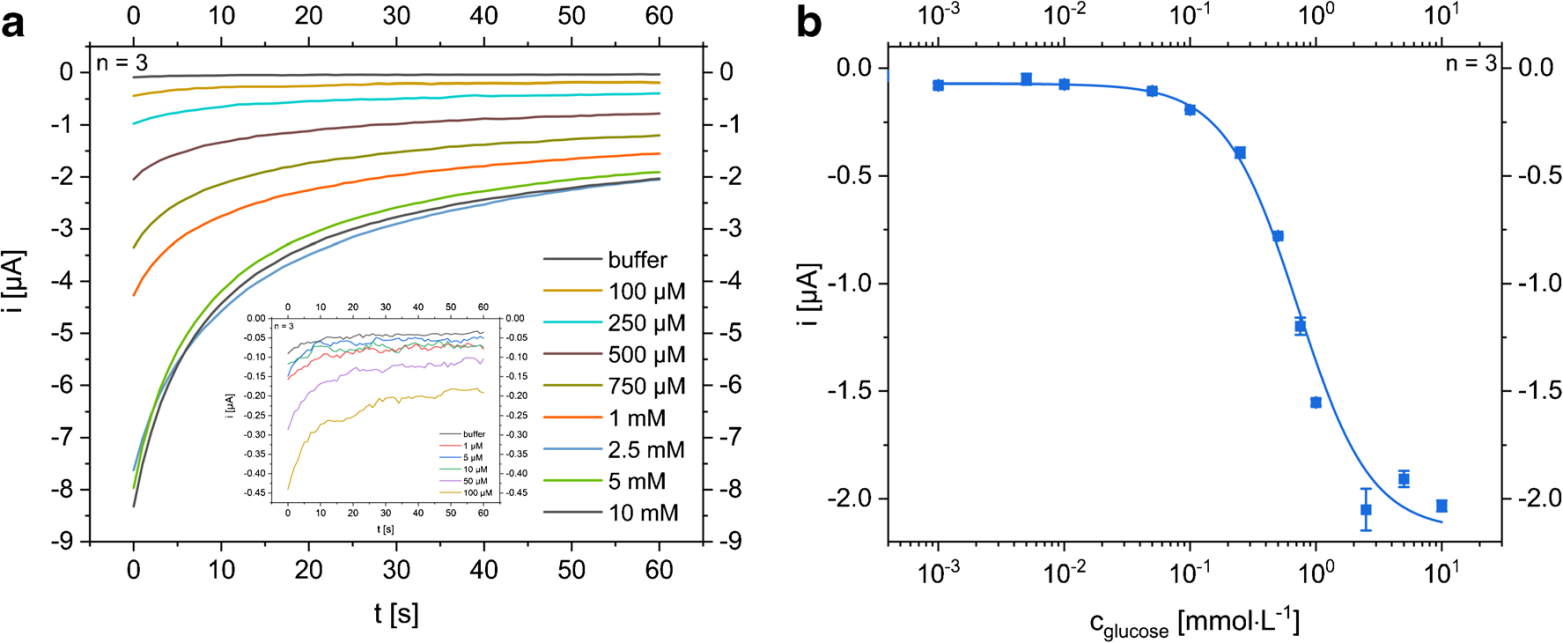
**a** Exemplary time versus current curves of chronoamperometric characterization of a modified glucose biosensor with chemical deposited Prussian blue layer, 0.1% chitosan membrane, and theoretical GOx activity of 1 U mm^−2^(*n* = 3, error bars are hidden for clarity). The applied potential was − 50 mV vs. LIG. Run time was 60 s and the sample was applied as a droplet. The magnified cutout demonstrates that low glucose solutions can be distinguished from each other. **b** Dose-response curve (*n* = 3, SD represented by error bars, droplet method) resulting from the respective I vs. t curve after a run time of 60 s on half-logarithmic scale. LOD is 13.7 ± 0.5 μmol L^−1^ and LOQ is 42 ± 2 μmol L^−1^

Enzyme-based biosensors were developed for the amperometric detection of glucose and lactate. Glucose oxidase and lactate oxidase were used as their reaction with the respective analytes produce H_2_O_2_, which can easily be detected on Prussian blue–coated carbon electrodes. Chitosan membranes were selected as protection membranes and scaffolds for enzyme immobilization due to their wide-spread and established performance in such enzyme sensors [1, 2, 31–37]. Here, these well-established concepts were established on the LIG electrodes. Enzyme immobilization, Prussian blue coating, chitosan membrane protection as well as acquisition and incubation time were optimized (see ESM Figs. S16–S20). For most of the optimizations steps, hydrogen peroxide (HP) sensors (without immobilized enzyme) were used and the characteristics of the underlying H_2_O_2_ sensor are shown in Table 1. Subsequently, the glucose sensor was tested in solution by dropping sample volumes onto the electrodes (Fig. 6), with a simulated sweat collection pad (wipe) and on chicken skin (Table 1). In the case of the droplet method, a limit of detection (LOD) of 13.7 ± 0.5 μmol L^−1^ and a limit of quantification (LOQ) of 42 ± 2 μmol L^−1^ were calculated from dose-response curves with an upper limit of the dynamic range around 2 mmol L^−1^ and a sensitivity of 20.0 ± 0.8 μA L mmol^−1^ cm^−2^_._ For the application intended, the detectable concentration range is appropriate, as glucose concentrations in sweat are in the range between 6 μmol L^−1^ and 2.2 mmol L^−1^ with a median value of 170 μmol L^−1^ reported [5].

**Table 1:** Comparison of important sensor characteristics determined with droplet method and sweat-collecting substrate for chronoamperometric detection of the respective analytes hydrogen peroxide, glucose, and lactate in 1× PBS pH 7.4 as well as in synthetic sweat solution according to DIN 53160-2 and artificial tear fluid with droplet application

Analyte	H_2_O_2_	Glucose		Lactate			
Sample application via	Droplet	Droplet	Filter on skin	Droplet	Synthetic sweat solution droplet	Artificial tear fluid droplet	Gauze
LOD (μmol L^−**1**^)	121 ± 6	13.7 ± 0.5	120 ± 4	28 ± 3	25 ± 4	82 ± 1	133 ± 1
LOQ (μmol·L^−**1**^)	367 ± 2	42 ± 2	365 ± 11	86 ± 6	76 ± 11	249 ± 2	415 ± 3
Linear range up to (mmol L^−**1**^)	5	1.5	1.5	1	5	1	2.5
Sensitivity (μA L mmol^−**1**^ cm^−**2**^)	3.0 ± 0.1	20.0 ± 0.8	4.5 ± 0.1	16 ± 1	6.1 ± 0.4	4.5 ± 0.6	1.75 ± 0.01
adj. *R*^2^	0.9736	0.9860	0.9895	0.9633	0.9377	0.9989	0.9864

In the case of filter paper and chicken skin analyses, LOD and LOQ are increased by a factor of nine to 120 ± 4 μmol L^−1^ and 365 ± 11 μmol L^−1^, respectively. The sensitivity of the sensor decreased by a factor of five to 4.5 ± 0.1 μA L mmol^−1^ cm^−2^ (ESM Fig. S21 b). We therefore suggest to change the sweat collection to other strategies in the future, such as macro-collection channels as published by Lei et al. [38], as also indicated by further CV analyses (ESM Fig. S21 a). This strategy would provide better analyte diffusion profiles and hence create conditions similar to those established in the droplet method.

**Fig. 7:**
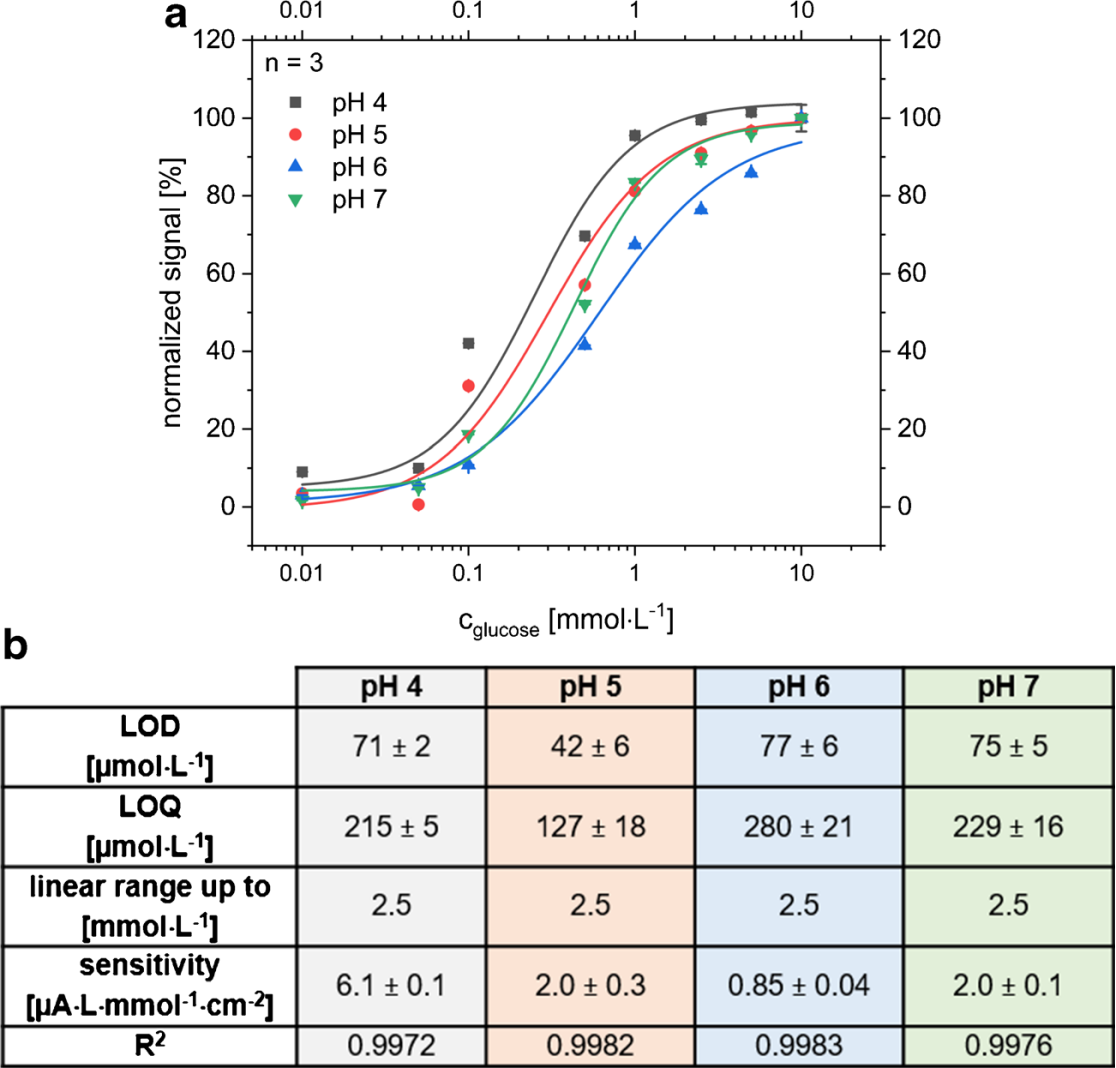
**a** Normalized dose-response curves of glucose in phosphate/citrate buffers at different physiological-relevant pH values (between 4 and 7). For each pH value, a new sensor was taken. A potential of E = 0 V vs. LIG was applied for all chronoamperometric measurements. All curves have a highly comparable shape independent of pH. Normalization of the signals from different sensors is necessary due to the varying sensitivity of the manually fabricated sensors. **b** Summary of important characteristics for the glucose sensors at different physiological-relevant pH ranges

Due to the broad pH range of sweat based on different factors like collection point, extent of activity, or fitness level of the individuals [6, 39], dose-response curves for glucose were recorded between pH 4 and pH 7. Each curve was measured on one sensor with an applied potential of 0 V vs LIG. The resulting dose-response curves and the calculated sensor specifications are comparable (Fig. 7). The dose-response curves were normalized due to the varying sensitivities of the different sensors. If the manual modification steps are replaced by an automated method, we assume that the reproducibility of the sensitivity can be increased. Currently, a two-point calibration for the lowest and highest concentration and normalization is a satisfying procedure for comparison.

Upon simply exchanging the enzyme from glucose to lactate oxidase, the strength of the sensor concept became obvious. No further optimization experiments were required. The lactate detection in the droplet format was performed within a concentration range from 10 μmol L^−1^ to 5 mmol L^−1^. A LOD of 28 ± 3 μmol L^−1^ and a LOQ of 86 ± 8 μmol L^−1^ were obtained with a sensitivity of 16 ± 1 μA L mmol^−1^ cm^−2^ (Table 1). Interestingly, the sensor is too sensitive for lactate concentrations in sweat, which ranges from 3.7 up to 50 mmol L^−1^ [5, 40]. This high level is a well-known challenge for oxidase-based biosensors in literature [5, 29, 41]. To overcome this challenge, perforated membranes could be used as demonstrated previously by our groups [38]. Experiments with gauze for sweat collection showed the same trend as previously seen for glucose detection. LOD and LOQ declined nearly by a factor of five compared with the droplet method to 133 ± 1 μmol L^−1^ and 415 ± 3 μmol L^−1^, respectively, with a sensitivity of 1.75 ± 0.01 μA L mmol^−1^ cm^−2^. In the case of lactate, the LIG-lactate sensor performs well within the lower physiological-relevant range. At the same time, materials other than gauze should be investigated in the future such as specialized polymer sponges, hydrophobic fibers, or microfluidic channels [41–46].

In the case of lactate as analyte, measurements in synthetic sweat matrix (DIN 53160-2) and in artificial tear fluid were also performed using the droplet method. Data plots for lactate measurements are shown in Fig. S22 (see ESM). The values of sensitivity, LOD, and LOQ are summarized and compared with all other amperometric determinations in Table 1.

The enzymatic sensors show at least comparable or even better LOD/LOQ and sensitivity than the underlying HP sensor. As the HP is produced directly on the sensor surface in the case of the enzyme sensors, dependence on HP diffusion is minimized and therefore the sensitivity can be improved. The sensor’s performances prove their applicability for the detection of glucose and lactate in the respective fluids representing real samples. While LOD and upper detection limit are shifted to higher values in comparison with their performance in buffer solution, the sensors are a suitable platform for quantification within the physiological range of lactate in eccrine sweat (3.7–50 mmol L^−1^ [5]) and tear fluid (1 to 5 mmol L^−1^ [47]). As indicated in Table 1, various filter papers were studied to function as sweat collection material. Depending on the ability to hold aqueous solution on top of the sensor surface, more or less hindrance of the detection itself was observed. Materials similar to simple filter paper work well in most instances as indicated by the filter paper, Kimwipe tissue, and gauze data shown. Moreover, all favorable data obtained from the single-parameter sensors suggested combining the sensors to a miniaturized POC multi-analyte sensing platform for a reliable and affordable sweat analysis.

### Combined multi-analyte sensor

**Fig. 8:**
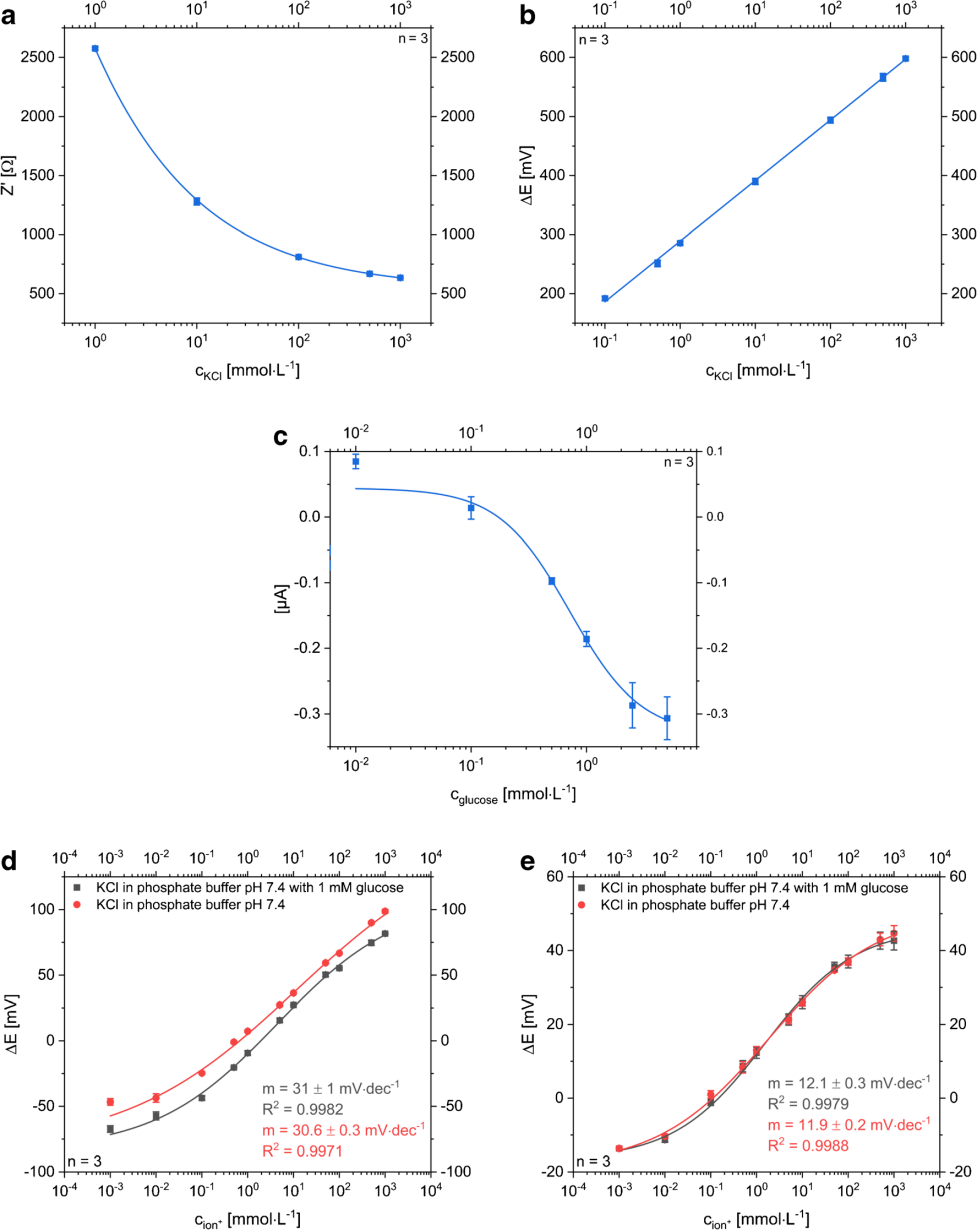
Dose-response curves of combined multi-analyte sensor for three determination methods under the same conditions as for the single-analyte sensor. All techniques show a comparable response as the single-analyte sensors. **a** The all-electrolyte concentration with the impedance measurement is highly reliable in the physiological-relevant range from 1 to 500 mmol L^−1^. **b** A similar reliability within the relevant range is provided for the detection of potassium concentrations with the potentiometric sensor. The slope of the linear fit is 103 ± 1 mmol dec^−1^. **c** Glucose determination with the amperometric setup. LOD of 135 ± 5 μmol L^−1^ and LOQ of 410 ± 15 μmol L^−1^ were obtained. Potentiometric determinations were performed with a **d** Nafion/KCl/PVC-protected RE and a **e** LIG RE

For the multi-analyte concept, the first challenge was to minimize the sensor area to provide reliable responses with analyte volumes as low as possible. Therefore, the Ag/AgCl reference electrode is successfully shared by the potentiometric and the amperometric setup (Fig. 2c and d). Cross talk of the electrodes is minimized by choosing a semi-simultaneous measurement approach in which sensors are turned on and off consecutively within a short time. Each analytical cycle (potentiometry, impedance, and chronoamperometry) requires only 75 s, and hence allows a quasi-continuous and simultaneous monitoring of all analytes. In this format, dynamic ranges and LOD/LOQ are comparable with the characteristics obtained for the single-parameter sensors (Fig. 8). This indicates that the changed layout and electrode geometry do not affect the performance. Most interestingly, it proves that multi-analyte sensing in an all-LIG format using any of the desirable electrochemical techniques can be realized and easily adapted to other analytes of interest to which ionophores or enzymes are available.

Table 2 shows similar sweat-sensing platforms in comparison with important key characteristics of our presented work. Our all-LIG multi-analyte sweat sensor scores well or even better than most comparable works, especially with respect to the simplicity of the fabrication process and the flexibility of introducing new analytes by changing ionophores or enzymes.

**Table 2:** Overview of comparable sweat-sensing devices (single-analyte and multi-detection systems) in comparison with our work

Analyte	Detection method	Electrode material	LOD	Detection range	Sensitivity	Response time	Year
Glucose	Amperometric, enzymatic (GOx)	rGO on PI with Au/Pt NPs + chitosan	5 μM	0–2.4 mM	48 μA/mM cm^2^	20 s	2018 [48]
Glucose	Amperometric, enzymatic (GOx)	SPE, graphite/Ag in microfluidic PET patch	11 μM	50–200 μM	1 nA/μM	1 min	2019 [49]
K^+^	Potentiometric, ISE (valinomycin)		–	5–40 mM	51.3 mV/dec	–	
Glucose	Amperometric, enzymatic (GOx)	Photo lithography on flexible PET; Ag/AgCl RE and CE, PB/chitosan/CNT on WE; PVB-RE, carbon/PEDOT:PSS	–	0–200 μM	2.35 nA/μM	1 min	2016 [29]
K^+^	Potentiometric, ISE (valinomycin)		–	1–32 mM	61.3 mV/dec	–	
Lactate	Amperometric, enzymatic (LOx)		–	0–30 mM	220 nA/μM	1 min	
Lactate	Amperometric, enzymatic (LOx)	SPE, Ag/AgCl, CNT, TTF, chitosan, on temporary tattoo	–	Up to 30 mM	10.31 μA/mM cm^2^	1 min	2013 [50]
Lactate	Amperometric, enzymatic (LOx)	SPE, PB/graphite, Ag/AgCl, carbon, chitosan	0.39 mM	0–14 mM	–	30 s	2017 [34]
K^+^	Potentiometric, ISE (valinomycin)		10^–3.9^ M	0–100 mM	58.0 mV/dec	20 s	
Glucose	Amperometric, enzymatic (GOx)	LIG, PtNPs, chitosan	< 300 nM	Up to 2.1 mM	4.622 μA/mM	–	2020 [51]
Glucose	Amperometric, enzymatic (GOx/LOx)	All-LIG-based, drop-coated/electrodeposited/chemical deposited Ag/AgCl RE, LIG RE possible, PB/chitosan WE	13.5 μM	Up to 1.5 mM	20.0 μA/mM cm^2^	60 s	This work
Lactate			28 μM	Up to 5 mM	16 μA/mM cm^2^		
K^+^	Potentiometric, ISE (valinomycin)		10^–4.5^ M	Up to 1 M	96 mV/dec	1 s	
Electrolytes	Impedance		10^–3.5^ M	1 mM–1 M	–	5 s	

*GOx*, glucose oxidase; *LOx*, lactate oxidase; *ISE*, ion-selective electrode; *PI*, polyimide; *rGO*, reduced graphene; *NPs*, nanoparticles; *SPE*, screen-printed electrodes; *PET*, polyethylene terephthalate; *RE*, reference electrode; *CE*, counter electrode; *WE*, working electrode; *PB*, Prussian blue; *CNT*, carbon nanotubes; *PVB*, polyvinyl butyral; *PEDOT:PSS*, poly(3,4-ethylenedioxythiophene) polystyrene sulfonate; *TTF*, tetrathiafulvalene; *LIG*, laser-induced graphene

## Conclusion

Laser-induced graphene (LIG) is a reasonably new, alternate graphene-like 3D carbon material investigated for electrochemical sensing, supercapacitors, and fuel cells [21, 27, 52]. Its simple fabrication requires a polyimide foil (such as commercially available Kapton sheets used herein), and a CO_2_ laser [24]. No additional pastes, substrates, gas environments, cleanroom conditions, or special know-how are needed, which catapults LIG electrodes into a category of most-easy-to-prepare electrochemical transducers from small lab-scale to large-scale production, easily amenable also to roll-to-roll fabrication. In this study, we demonstrated an all-LIG multi-analyte-sensing platform, employing the three relevant electrochemical principles used in point-of-care sensors (i.e., voltammetry, potentiometry, impedance) and addressing a relevant analytical challenge by applying it to sweat analysis. The inherent mechanical flexibility of the LIG substrate paired with the electroanalytical performance of the 3D graphene-like network enabled the detection of all chosen analytes (electrolyte, potassium ion, glucose, and lactate) in their relevant physiological range. Furthermore, most recent work demonstrated the non-toxicity of the LIG electrodes [53] and previous studies using aptamers indicated that highly sensitive bioanalytical sensors are feasible [21, 54]. Interestingly, when using more advanced substrates, such as polyimide nanofiber mats, new strategies are possible that create nanoparticle-embedding LIG nanofibers [55]. Similar strategies may be feasible when generating polyimide-nanoparticle blends and spin-coating those onto flat surfaces prior to pyrolysis via the laser. We predict that LIG electrodes will play a major role in future electroanalytical systems, not only applied to the point-of-care, but also to other low-cost analytical challenges such as food and environmental monitoring, since the all-LIG-sensing platform is feasible and can easily be fabricated even under resource-limited conditions.

##  Electronic supplementary material

ESM 1 (PDF 1968 kb)
