# Supplementary material for: Electrochemical multi-analyte point-of-care perspiration sensors using on-chip three-dimensional graphene electrodes
Source: Anal Bioanal Chem. 2020 Sep 28;413(3):763–77. doi: 10.1007/s00216-020-02939-4 (PMC7809000; doi:10.1007/s00216-020-02939-4)
Supplement: Supplementary file 1 — (PDF 1968 kb) [file 216_2020_2939_MOESM1_ESM.pdf]

Analytical and Bioanalytical Chemistry

Electronic Supplementary Material

Electrochemical multi-analyte point-of-care perspiration sensors using on-chip three-dimensional graphene electrodes

Meike Bauer, Lukas Wunderlich, Florian Weinzierl, Yongjiu Lei, Axel Duerkop, Husam N. Alshareef, Antje J. Baeumner

Laser-Scribing Process

The general laser-scribing process is schematically shown in the following Fig. S1. Flexible PI substrate is exposed to a commercial infrared CO_2 laser. The desired pattern can be “printed” on the substrate using suitable power and speed settings to obtain the porous graphene-like material.

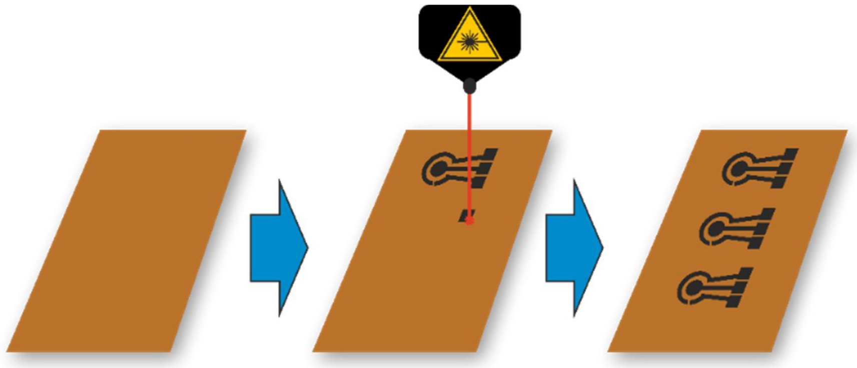

Fig. S1 Scheme of the laser-scribing process: a polyimide foil (orange) as flexible substrate for the desired pattern is exposed to a commercial infrared CO_2 laser (λ = 10.6 µm) with suitable laser power settings and scribing speed under ambient conditions to provide a porous 3D graphene material (black) with properties appropriate for electrochemical analysis

LIG electrodes produced with the VLS 2.30 laser scriber show different performance depending on the orientation during the scribing process. The scribing direction is always along the Y-axis, the laser pathway is always perpendicular to the scribing direction along the X-axis. Primitive designs were created that consisted only of two contact pads connected by a conductive strand. A design with a linear connection was produced; once orientated perpendicular to the scribing direction, once rotated by 45°, and another one parallel to the scribing direction. (Fig. S2 a) The resistance of the designs (N = 3) was measured from the middle of one pad to the middle of the other pad with a commercial voltmeter (distance of 5 cm).

While orientated in scribing direction, the resistance was 594 ± 27 Ω and therefore the lowest. If produced in a 45° angle, an increase in resistance by 76 % could be observed (1048 ± 13 Ω). Production perpendicular to the scribing direction lead to an increase by 128 % (1353 ± 37 Ω).

All structures that are not aligned to the scribing direction increase the resistance of an electrode significantly. A second set of designs was created with the same length and thickness as before but once with a 90° bend and once with the kink smoothed to a curve. (Fig. S2 b) The design with the 90° bend showed an increase in resistance by 65 % (982 ± 19 Ω), introducing the curve lead to an increase of 54 % (916 ± 9 Ω).

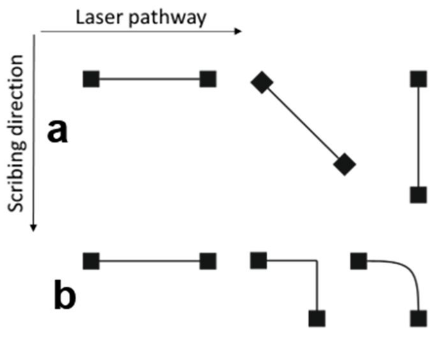

Fig. S2 **a** linear designs for resistance measurements; **b** designs with kink or curve.

Optimization and Modification Process for Potentiometric Potassium Ion Sensor

To prove the measurement principle, at first a simple potentiometric setup was tested in bulk solution by using the ion selective membrane modified LIG electrode (diameter d = 2.5 mm) versus a commercial external Ag/AgCl electrode. As can be seen in Fig. S3, the dose-response curve shows the expected trend over a large concentration range. The slope of 51 mV is quite close to the theoretically expected value of 59 mV. Changes in ionic strength did not influence the potential between an unmodified LIG electrode and the commercial Ag/AgCl RE.

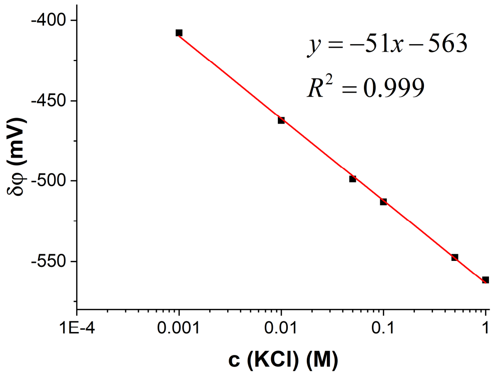

Fig. S3 Dose-response curve of a LIG electrode with a valinomycin doped membrane, using a standard Ag/AgCl electrode as reference in bulk solution. Coherence which shows LIG potassium selective electrode is suitable (n = 1)

Since the initial measurements were performed by dipping the electrodes into solution, the setup was changed to a planar LIG electrodes on one flexible substrate. To enable a planar electrode setup a new design had to be found Therefore, both electrodes had to be closer together, to enable a full coating of both electrodes with just one drop of sample solution. The setup used for measurements shown in Fig. S4 is the best compromise between analogy to the real wearable setup and working convenience.

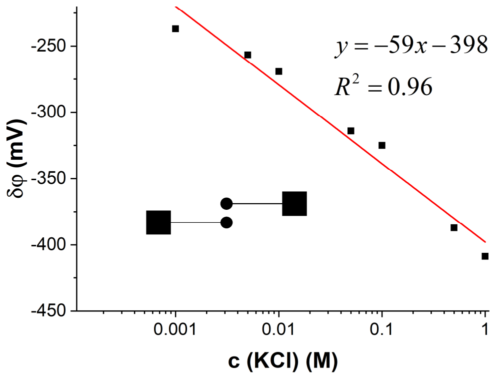

Fig. S4 Dose response curve of a valinomycin coated electrode versus a planar Ag/AgCl-coated LIG reference electrode. The corresponding electrode design is shown as inset. The measurements were performed by depositing a drop of potassium chloride solution on the planar electrode. Since the curve show a linear behavior the measurement principle was approved working (n = 1)

The thickness of the ion-selective membrane was about 70 µm (measured by optical light microscopy) and the concentration of the ionophore in the membrane solution was 16 mg per gram PVC. Both parameters might have potential to be optimized, but as the slope of the calibration curve is close to the theoretical value no other combinations have been tested. Furthermore, valinomycin selectivity towards K^+ ions was demonstrated against to the main interfering cations Na^+, Mg^{2+} and Ca^{2+} as shown in Fig. S5. Potassium ion concentration was constant at 10 mmol·L^{-1} whereas the interfering cation concentrations were varied between 0.1 and 50 mmol·L^{-1}.

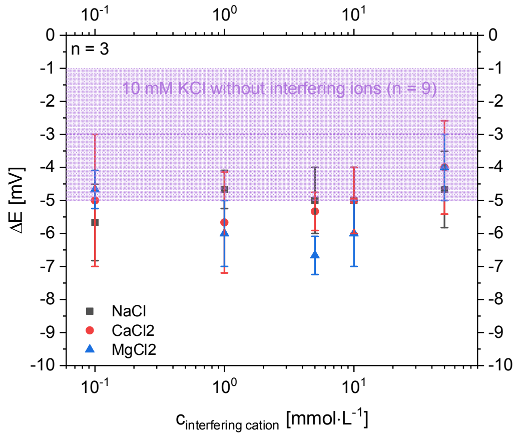

Fig. S5 10 mM KCl solution in water were measured (mean value with standard deviation is shown as violet box, n = 9). 10 mM KCl solutions with main interfering cation concentrations between 0.1 and 50 mM were measured. They show a small signal decrease to the sample solution without interfering ions. **Grey boxes** NaCl **red circles** $CaCl_2$ **blue triangles** $MgCl_2$ solutions were measured with the planar design using the drop-coated ISE vs. unmodified LIG electrode

The dose-response curve reveals that the principle will work on LIG electrodes. The signal changes upon changing the concentration were measured immediately after sample application and after 60 seconds with the silver paste based Ag/AgCl RE. Both resulted in stable and reproducible values (Fig. S6).

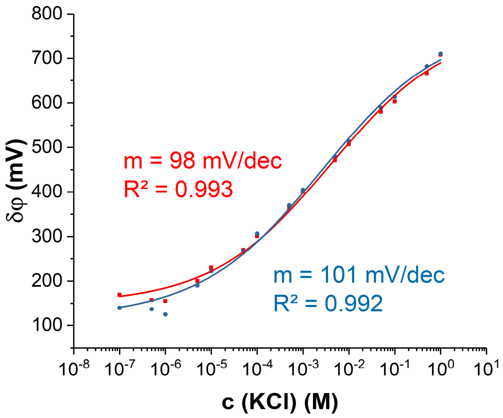

Fig. S6 Dose-response-curve the potentiometric, planar LIG based sensor, measuring a KCl concentration ranging from 10^{-7} to 1 M. In this case the reference electrode was synthesized using a conductive silver paste (n = 3). The linear range goes down to 10^{-4} M KCl. The red curve shows the potential directly after sample application with a slope of 98 mV per decade, whereas the blue curve shows the potential after 60 s with a slope of 101 mV per decade, respectively. For both times the curves show good coherence

As a next step, to further improve the LIG based electrode setup, a different planar reference electrode was used. Up to now a conductive silver paste with deposited silver chloride was used (Fig. S7 a), which was suitable for initial measurements, but since the deposition of this paste cannot be performed reproducibly and it is not known which additional ingredients are contained in this paste, the reference electrode needed further improvement.

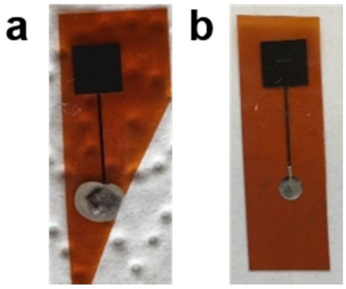

Fig. S7 a LIG based reference electrode, using a conductive silver paste. **b** Ag/AgCl RE which was made by using a plating device and $AgNO_3$ electrolyte. The improved procedure, using a plating device, enables a much more reproducible fabrication process

Therefore, first Ag should be electrodeposited with from a silver nitrate solution and, in a second step, the surface layer oxidized to silver chloride. A commercially available handheld plating device was used for deposition with a $AgNO_3$ solutions, mass concentration ranging between 200 mg·mL^{-1} and 1000 mg·mL^{-1}. One drop of electrolyte, between 10 and 50 µL, was deposited on the LIG. For a uniform deposition it was needed to equilibrate the silver nitrate solution on the LIG. Different equilibration times between 1 minute and 15 minutes were tested and the uniformity after the plating process was evaluated by naked eye. The actual plating was executed between a few seconds and 2 minutes. An immediate formation of a silver layer on the LIG could be observed. To form the silver chloride coating, the silver-plated electrode was immersed into a saturated KCl solution and a DV voltage of 800 mV was applied for 60 seconds up to 200 seconds. A slight darkening of the silver layer could be seen (Fig. S7 b).

In a 10^{-2} M KCl solution this new planar LIG based reference electrode shows a potential of 2 mV versus a silver wire coated with silver chloride, which indicates that the coated LIG electrode should be suitable as reference electrode. As can be seen in Fig. S8 the improved LIG-based RE decreases the linear range of the potentiometric potassium selective sensor down to 10^{-5} M KCl.

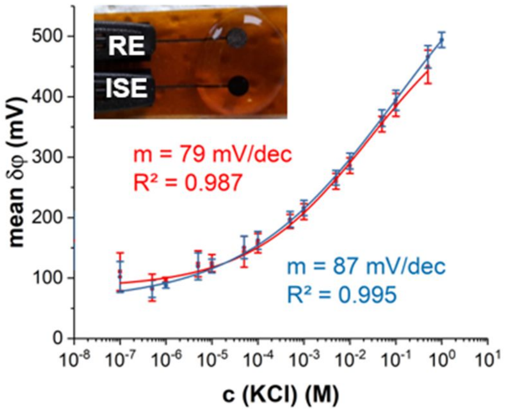

Fig. S8 Dose-response-curve the potentiometric, planar LIG based sensor, measuring a KCl concentration ranging from 10^{-7} to 1 M. In this case the Ag/AgCl RE was electrodeposited with a plating device. The sensor design is shown as inlet. The linear range goes down to 10^{-5} M KCl. The red curve shows the potential after 0 s with a slope of 79 mV per decade, whereas the blue curve shows the potential after 60 s with a slope of 87 mV per decade, respectively. For both times the curves show good coherence

Finalized parameters are summarized in the experimental section. The final modification procedure to obtain a potentiometric LIG sensor is shown in the following Fig. S9.

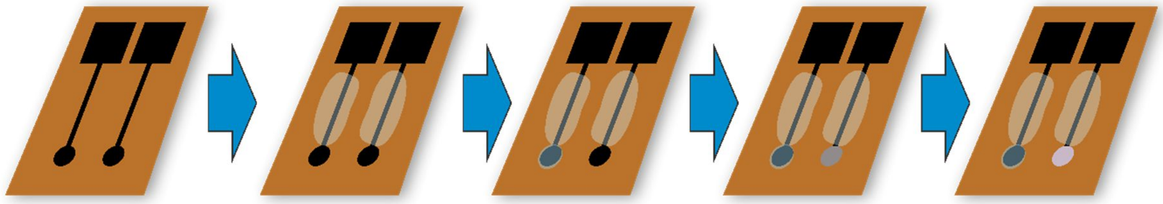

Fig. S9 The entire modification process for the potentiometric sensor is shown schematically from left to right starting with the raw LIG pattern, followed by the insulation of the strands and drop-coating of the ion-selective membrane cocktail on the left electrode. Afterwards, on the right electrode, a silver layer is plated and in the last drawing the formed AgCl layer is shown

Optimization of Impedance-based Electrolyte Sensor

Cell constants for the interdigitated structure are calculated from the ratio of the space S between two fingers and the width W of one finger. According to literature [56] an optimized cell constant is obtained for a S/W ratio of 0.54. Furthermore, the length L and the number N of the electrode fingers are important characteristics, and both should be as large as possible to improve the cell constant. In a first attempt, L was set to 6 mm and N was set to 10, which results in a total electrode size of approximately 15 mm height and 8 mm width.

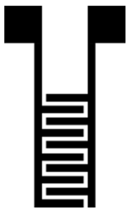

Fig. S10 Layout of an interdigitated Laser-Induced-Graphene electrode on polyimide substrate

The electrode layout needed further improvements with regard to respective scribing conditions. Right angles and lines perpendicular to the scribing direction should be avoided as explained above. The evolution of the layout is shown in Fig. S11 where we finally ended up with the kidney-shaped design which was used for all investigations in the main part. For all designs, the line width of the electrodes is 0.5 mm and spacing between the electrodes is 0.2 mm.

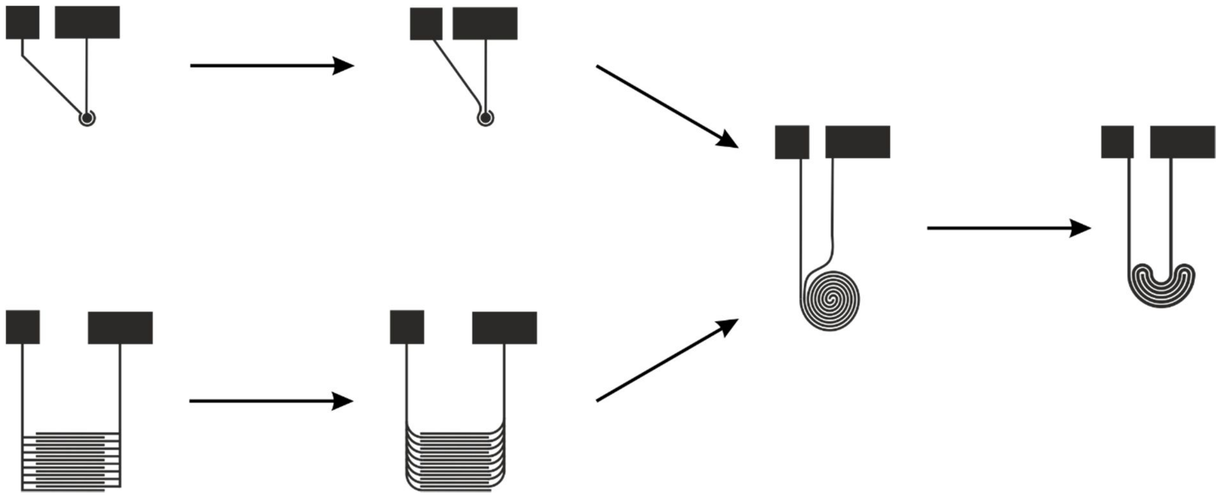

Fig. S11 Optimization of LIG electrode layout for impedance measurements. Since right angles increase the electrode resistance, the design was created with the least numbers of right angles. It turned out, that an interdigitated design with a higher electrode area showed better results. Horizontal structures show decreased resistance since they are orientated in the scribing direction

The in following described optimization procedures were performed with the previously developed spiral design (Fig. S12 a). These procedures are concerning especially the instrumental settings and sample application techniques. For the measurements shown in the main part of the work the kidney-shaped design (Fig. S12 b) was used.

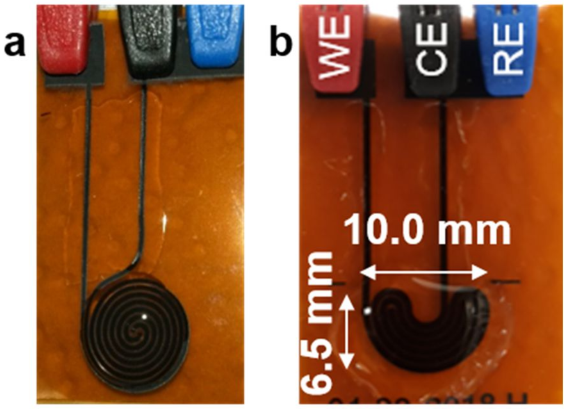

Fig. S12 a Spiral electrode design for impedance measurement as previous version of the finally used kidney-shaped design shown in **b.** Line width is 0.5 mm; line spacing between the electrodes is 0.2 mm

A full impedance spectrum of a LIG electrode was measured at E_{DC} = 0 V, E_{AC} = 0.1 V, from 100 kHz to 1 Hz for a 0.5 M NaCl solution (Fig. S13 a). The full spectrum is compared to a measurement at a fixed frequency at 1000 Hz (Fig. S13 b). It can be seen, that the impedance value of the fixed frequency matches the value obtained out of the full spectrum. Measuring at a fixed frequency of 1000 Hz reduces sensor's response time from minutes to a few seconds.

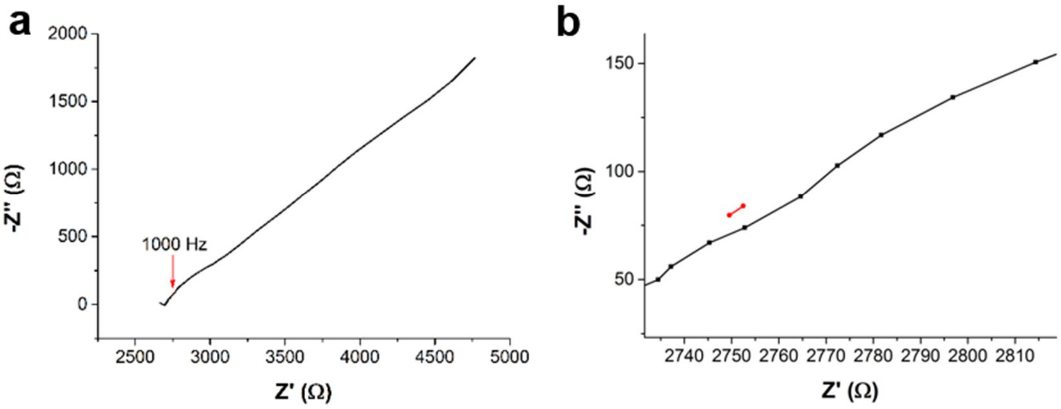

Fig. S13 Spiral design was used for all measurements. **a** Impedance spectrum of a LIG electrode measured at E_{DC} = 0 V, E_{AC} = 0.1 V, from 100 kHz to 1 Hz immersed into a 0.5 M NaCl solution. **b** Comparison of the full spectrum (black, magnified cut-out from a) with a measurement at a fixed frequency of 1000 Hz (red). It can be seen, that the impedance value of the fixed frequency matches the value obtained out of the full spectrum

Next, the dose-response curves are shown. NaCl solution in a concentration range between 28 and 200 mM were measured. The data shown in Fig. S14 a were derived from a full spectrum measurement. The values were taken at a frequency of 1 kHz. Data shown in Fig. S14 b were obtained from a measurement of the same samples at fixed frequency of 1 kHz. There is no significant difference between both. A measurement at fixed frequency is much faster and therefore preferable.

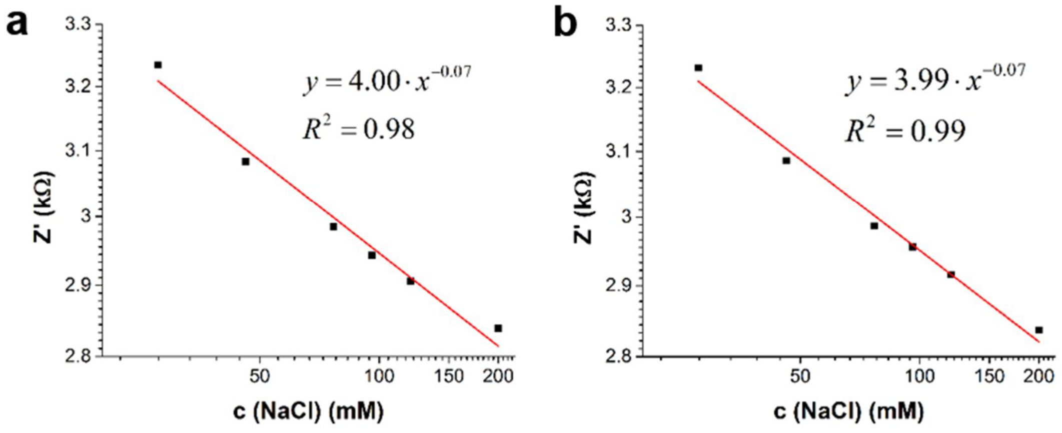

Fig. S14 Dose-response-curves of LIG electrodes, measured at E_{DC} = 0 V, E_{AC} = 0.1 V, from 100 kHz to 1 Hz for NaCl concentrations ranging from 28 mM to 200 mM. Impedance values at 1000 Hz are taken from **a** a full spectrum and **b** a measurement at fixed frequency was performed with the same samples

Fig. S15 a shows full impedance spectra, measured at E_{DC} = 0 V, E_{AC} = 0.1 V, from 100 kHz to 1 Hz for a 0.2 M NaCl solution. The black spectrum was measured by immersing the electrode into solution, whereas for obtaining the red spectrum a drop of salt solution was deposited on the electrode structure. Fig. S15 b shows the corresponding dose-response-curves for NaCl concentrations ranging between 28 mM and 200 mM. The impedance value was taken out of the full spectra at 1000 Hz. The curves do not match; therefore, one application mode must be chosen.

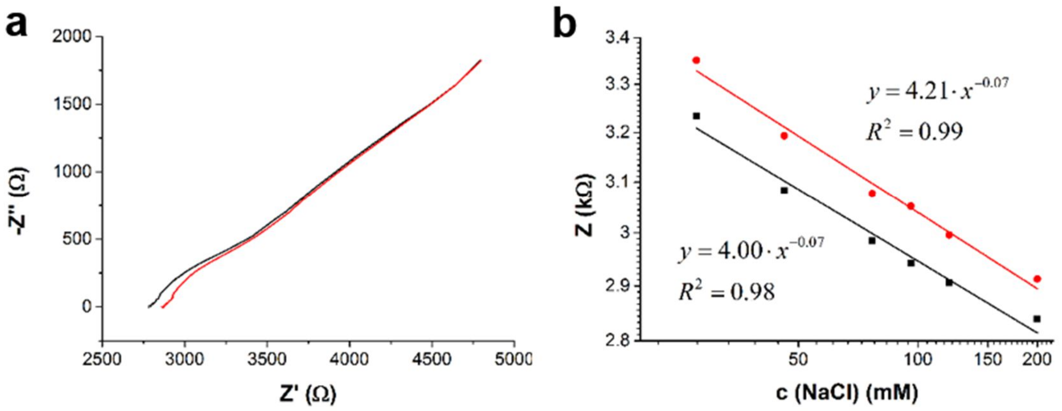

Fig. S15 a Impedance spectra, measured at E_{DC} = 0 V, E_{AC} = 0.1 V, from 100 kHz to 1 Hz for a 0.2 M NaCl solution. The black spectrum was measured by immersing the electrode into solution, whereas for obtaining the red spectrum a drop of salt solution was deposited on the electrode structure **b** Corresponding dose-response-curves for NaCl concentrations ranging from 28 mM to 200 mM. The impedance value was taken out of the full spectra at 1000 Hz. The curves do not match; therefore, one mode of application should be chosen

Optimization of Amperometric Biosensors

Two different methods for PB deposition were investigated [57, 58]. Subsequently, differently concentrated chitosan solutions for drop-coating a suitable membrane were applied using literature conditions [35, 59]. Furthermore, the electrodeposited and well-reproducible Ag/AgCl RE was compared to a LIG reference electrode. Reproducibility and complexity of production were also taken into account for the decisions. During these fundamental optimization steps a suitable run time for the amperometric measurement was determined, which meets the requirements with regard to accuracy and time effort. Last step was optimization of enzyme immobilization and incubation time.

Electrodeposition of Prussian Blue

An often-reported method for PB deposition on a graphene or carbon-based surface is the electrochemical deposition using cyclic voltammetry [58, 60]. The electrodeposition was investigated with different experimental setups and electrodes. In the beginning a vertical three-electrode setup with an external Ag/AgCl RE and a platinum wire CE was used. Later, the electrodeposition was performed in the planar all-LIG setup with a LIG reference electrode and the self-made electrodeposited Ag/AgCl RE, respectively. Different scan speeds and number of cycles were investigated.

First, the planar sensor with the electrodeposited Ag/AgCl reference electrode was tested. Depositing PB solely on the LIG WE was not possible using the sensor layout. After drop-coating the PB precursor solution, without applying a potential or current, a redox reaction started forming a clearly visible blue PB layer on the Ag/AgCl surface. Therefore, the sequence of electrode modification was swapped. The PB layer was deposited first in vertical setup using an external RE and in the second step the Ag/AgCl deposition procedure was performed. It is important to ensure the silver nitrate solution does not touch the PB layer due to the spontaneous formation of silver on the WE surface.

The unmodified sensor and both external electrodes were fixed and immersed into the deoxygenated and freshly mixed precursor solution. After a few CV scans, independent of scan rate and potential step width, a dark blue layer on the WE was recognizable with the naked eye. After at least ten cycles the PB layer was distinctive visible and furthermore PB was precipitated in the precursor solution. During removing the sensor from the setup and also during electrode cleaning with water, a large part of the blue layer dropped off. This vertical setup proved soon to be a laborious method where a large amount of the precursor solution is needed. Therefore, the planar setup was tested again with the unmodified LIG as reference electrode. Fig. S16 a shows the resulting CV scans during the electrodeposition process of Prussian blue versus a LIG reference electrode in a planar setup (Fig. S16 b). The resulting PB layer before cleaning with water is shown in Fig. S16 c.

Compared with literature data measured in conventional cells under similar conditions (pH, buffer, concentrations, instrumental settings), the shape of the single CVs and also the observation with increasing repetitions are similar [58, 60]. With every additional scan the peak currents increase whereas a slight potential shift, especially for the reduction peaks, is observed. The characteristic four main peaks of both redox couples Prussian blue/Prussian white and Prussian blue/Berlin green are clearly differentiable. For a reproducible sensor production and also with regard to instrumental effort, the electrochemical deposition of PB was not the best option. The formed PB layer was rather sensitive and even got damaged during rinsing to remove the excess PB precursor solutions.

Chemical Deposition of Prussian Blue

Ricci *et al*. described a simple procedure for the modification of screen-printed graphite electrodes via chemical deposition of PB [57]. The protocol was varied to increase the reproducibility of the redox mediator layer. Therefore, the PB precursor solutions are mixed exclusively on the WE area (d = 3 mm) and soaked in the porous graphene layer for 20 minutes at ambient conditions. Afterwards, the layer was stabilized and activated for 2 hours at 100 °C. To avoid an undefined loss of PB, the

chitosan membrane was drop-coated before the final washing step was performed. An image of the modified sensor was captured with a handheld digital microscope and is shown in Fig. S16 d. Due to the comparable performance of the sensors and the straightforward production process, which is also more compatible with the Ag/AgCl deposition, the chemical deposition was chosen.

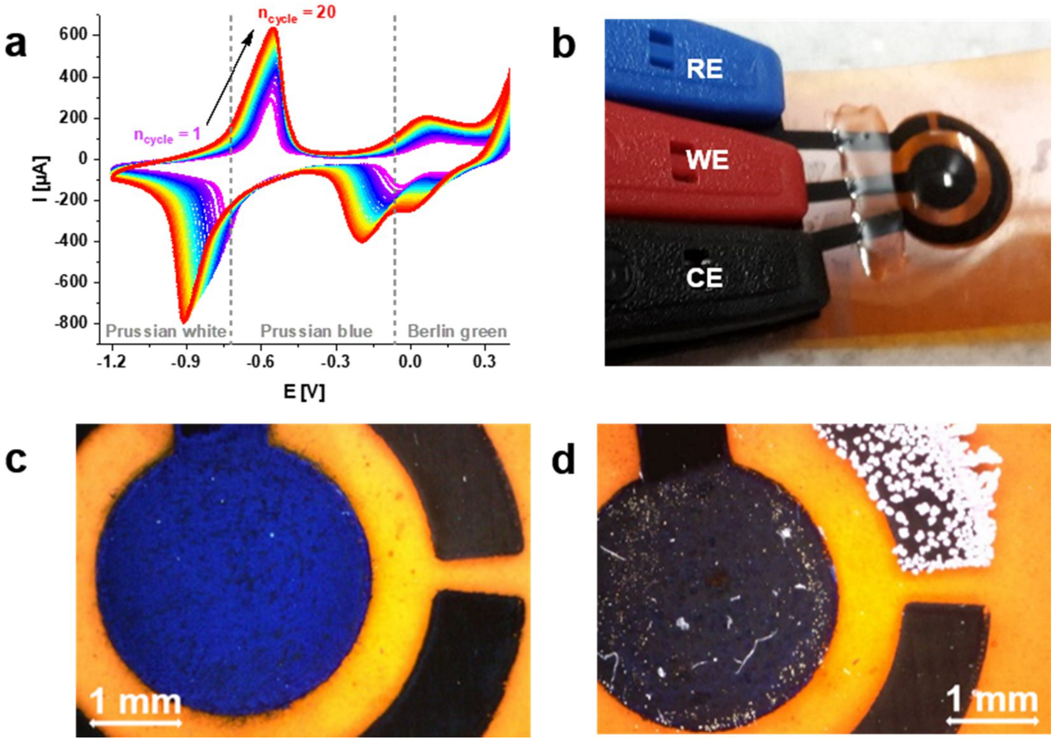

Fig. S16 a CV scans of Prussian blue electrodeposition on the LIG working electrode. The CV was performed from 0.5 to -1.2 V vs. LIG (20 mV·s^{-1}) in a planar setup (**b**) with applied PB precursor solutions. **c** The WE area modified with electrodeposited PB. The electrochemical deposition was used in a planar setup vs. LIG RE and the image was captured before washing steps. The LIG counter electrode is unmodified. **d** PB layer on the WE was chemically deposited. To avoid an undefined loss of PB, the chitosan membrane was drop-coated before the final washing step was performed. On the RE the Ag/AgCl layer was electrodeposited after PB deposition. The CE is unmodified. (The magnified images were captured with Dino Lite digital USB microscope with suitable software Dino Capture 2.0

Ag/AgCl and LIG as Reference Electrode Material

CVs of pure buffer were measured in the planar setup versus internal RE. The sensors were prepared with chemical deposited Prussian blue and 0.5% chitosan solution. Representative cyclic voltammograms are shown for both setups in Fig. S17. The whole CV versus Ag/AgCl is shifted by around 0.4 V to higher potentials in comparison to the LIG reference electrode. Using a LIG reference electrode is favorable because the sensor's working range is near 0 V. The peak currents are higher than the ones using the Ag/AgCl electrode that promises an increased sensitivity of the sensor. Furthermore, the practically nonexistent modification step pleads also for LIG as RE material. A large drawback of the LIG RE was the occurring potential shift during repeated CV scans as well as during the amperometric detection. Therefore, the RE modification is necessary.

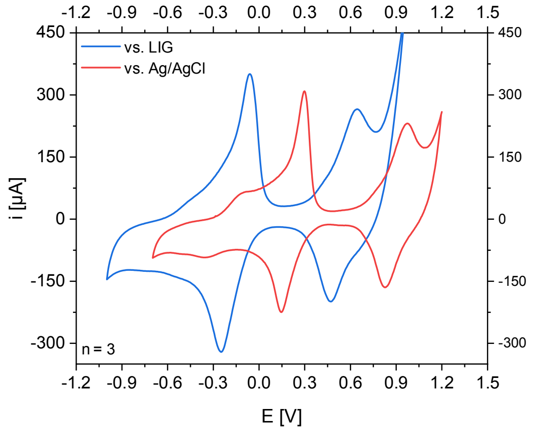

Fig. S17 Comparison of representative CVs (PBS; scan speed 50 mV·s^{-1}) were determined in a planar sensor setup with droplet method. The PB layer was chemically deposited and covered by a chitosan membrane (0.5% chitosan solution). CVs were measured versus internal LIG (blue) and versus electrodeposited Ag/AgCl reference electrode (red). Dependent on the RE used the peak potentials are shifted

Optimization of Chitosan Membrane Thickness

Same volumes of chitosan solutions with concentrations of 0.1%, 0.25% and 0.5% were applied to the WE area and dried overnight at ambient conditions. After buffer measurements, four H_2O_2 solutions in a concentration range from 10 to 500 µmol·L^{-1} were applied three times. Chronoamperometry was run for 60 s at the respective suitable potential. The resulting currents and the signal-to-noise ratio are shown and compared in Fig. S18. The sensor with the lowest chitosan concentration (0.1%) applied, showed for all hydrogen peroxide (HP) concentrations the highest current. It is plausible that the lower concentrated chitosan solutions result in a thinner membrane than higher concentrated ones when the same volume was applied. Therefore, the diffusion of the analyte but also interfering compounds, is less hindered in a thinner membrane. On the other hand, with the 0.5% chitosan membrane for HP concentrations of 50 µmol·L^{-1} and above, a significant increased S/N was obtained. The thicker membrane increases the selectivity but reduces the sensitivity. Experiments were continued with 0.1% and 0.5% chitosan membranes but subsequently the 0.1% chitosan solution was used solely. The later immobilized biorecognition element increases the selectivity and therefore the higher sensitivity of the thin membrane is preferable.

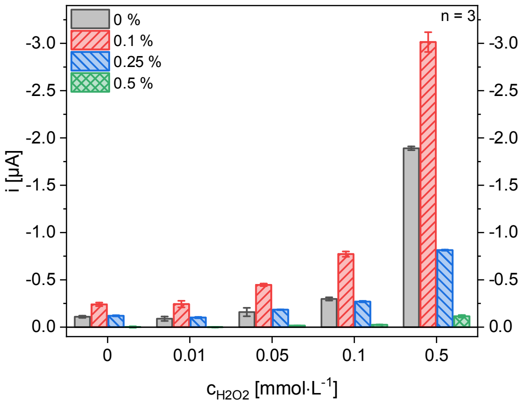

Fig. S18 Different concentrated chitosan solutions (0%, 0.1%, 0.25%, 0.5%) were drop-coated on the WE of hydrogen peroxide sensors with chemical deposited PB layer (n = 3). The signals, which were obtained 60 s after application of HP solutions in 1X PBS pH 7.4, were compared

Optimization of Chronoamperometric Response Time

During all the modification and optimization steps a suitable chronoamperometric run time was determined. Chronoamperometry was run for 180 s. Every 30 seconds the responded data were evaluated with regard to signal and principally stability. Results for 10 and 100 µmol·L^{-1} H_2O_2 solutions are shown in Fig. S19. The signal is almost stable after 60 seconds for 0.1 mmol·L^{-1} HP solutions. All signals of lower concentration are similar within the error bars. The standard deviations of the three-fold determinations are slightly decreasing for both H_2O_2 concentrations with increasing response times. With regard to real-time monitoring, a short run time is preferable. Therefore, 60 seconds acquisition time is a suitable compromise between response time and reliability of the signal.

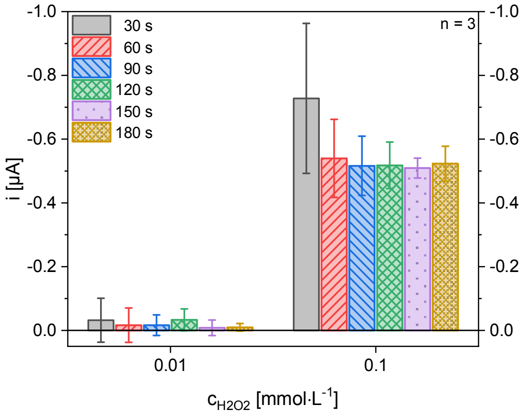

Fig. S19 10 and 100 µM hydrogen peroxide solutions were applied onto the sensor and the chronoamperometric measurements were run for 180 s (n = 3). Every 30 seconds the response data were evaluated. Signal and signal stability (refers to standard deviation) were considered to find a compromise between response time and reliability of the signal

Immobilization of Enzyme, Activity and Incubation Time Experiments

For the amperometric sensor, different enzyme immobilization strategies found in literature [29, 33, 34] were adapted and tested. The sensors' performances were compared, and the results are shown summarized in Fig. S20 a. A reason for failing of the immobilization strategies a) and b) is the acidic milieu of the chitosan solution. Chitosan is only soluble in acidic aqueous solution that is no suitable

medium for the sensitive enzymes over a longer period. Therefore, extended contact with the acetic solution probably inactivated or damaged the glucose oxidase. Applying the enzyme solution on top of the chitosan network, could lead to a loose entrapment within the polymer structure.

The same volume of GOx aliquots (theoretically calculated activity of 0.4 and 4 U·µL^{-1}) was applied onto the chitosan membrane. The resulting GOx activities therefore were 0.1 and 1 U·mm^{-2} on the working electrode, assuming a planar surface. Incubation times of 1 and 5 minutes were tested and the respective signals were compared (Fig. S20 b). To compare different sensors directly with each other, the signal of glucose was normalized to the signal of the respective H_2O_2 solution. As expected, higher enzyme activity within the same incubation time provided a higher signal (50 % higher for the 5 min incubation example shown in Fig. S20 b). Similarly, longer incubation resulted in higher signals. The incubation time experiment was repeated with 5 U·µL^{-1} LOx instead of GOx solutions (Fig. S20 c). Also here, longer incubation times provide a higher normalized signal. With regard to the later application as continuous sensing device long incubation times are not preferable. Therefore, the application of a high enzyme amount is favorable.

a

	immobilization strategy	performance
1)	enzyme covered with chitosan solution [34]	no measurable response
2)	enzyme premixed with chitosan solution [29]	no reproducible results
3)	enzyme applied onto chitosan membrane [33]	reliable signals

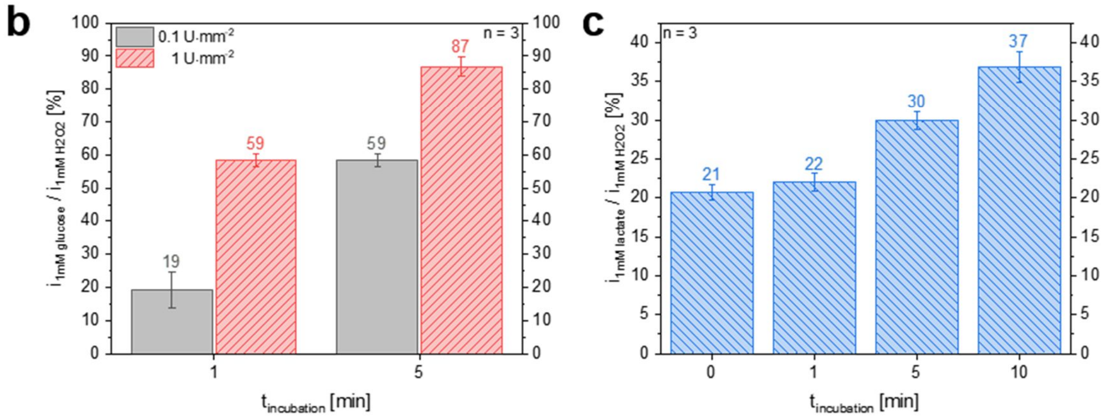

Fig. S20 a Different immobilization strategies with chitosan as enzyme-friendly material were adapted and tested. The sensor performances suggested an enzyme application on top of the chitosan layer **b** 2 µL of two different GOx solutions (0.4 U·µL^{-1} and 4 U·µL^{-1}) were applied on top of the sensors and incubated overnight at 4 °C. The resulting enzyme activities at the WE were calculated as 0.1 U·mm^{-2} (grey) and 1 U·mm^{-2} (red). The signals are normalized to the signal of 1 mM hydrogen peroxide solution **c** Enzyme incubation experiments were also performed with a 5 U·µL^{-1} lactate oxidase solution drop-coated on top of a chitosan/PB modified WE (each n = 3)

Glucose Biosensor Performance on Skin

To proof the sensors functionality on biological substrates, the experiments were repeated on chicken skin using filter paper as sweat collecting tissue. Detection on the blank chicken skin was not possible. Poor conductivity and a blocked electrode surface were observed, but the reasons therefore were not further investigated. With a sweat absorbing substrate layer between electrodes and chicken skin the sensors performance was recovered. A noticeable difference to the droplet method was the shift of sensor's suitable working potential to a higher potential as shown in Fig. S21 a. Especially, in the

anodic current region the peaks were distorted compared to CVs obtained with droplet method. Thus, the peak separations were significantly increased indicating a restricted electron transfer. Glucose concentrations between 1 µmol·L^{-1} and 10 mmol·L^{-1} were measured three times on chicken skin with filter paper at a slightly increased potential compared to droplet method. The resulting calibration curve is shown in Fig. 21 b. Due to the previous obtained results during potassium sensor characterization, higher LOD and LOQ were expected. Indeed, both values are increased by a factor of nine to 120 ± 4 µmol·L^{-1} and 365 ± 11 µmol·L^{-1}compared to droplet method. Sensor sensitivity decreased by a factor of five to 4.5 ± 0.1 µA·L·mmol^{-1}·cm^{-2}.

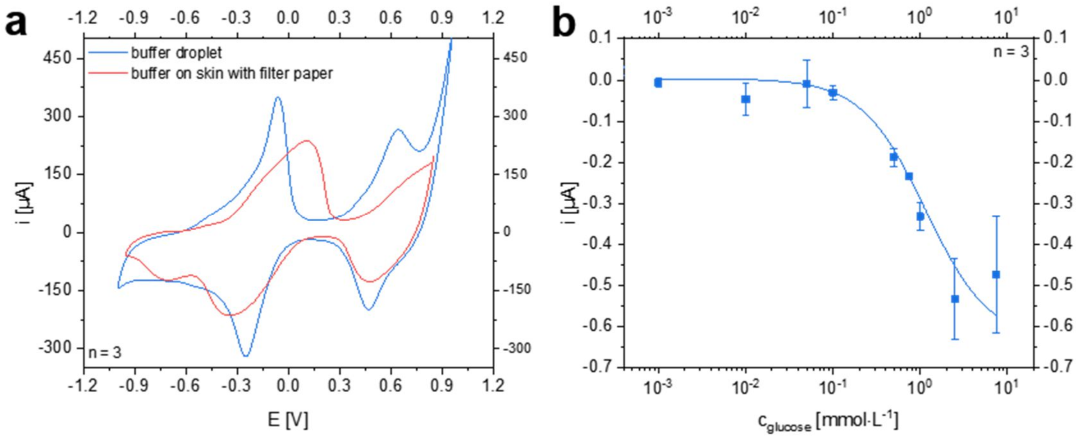

Fig. S21 a CVs of glucose sensor in buffer (50 mV·s^{-1} vs. LIG) with droplet method (blue) and measured on chicken with filter paper as sweat collection pad (red) **b** Dose-response curve (n = 3, SD represented by error bars) of amperometric detection of glucose on chicken skin with filter paper as sweat collection pad at a potential of 0.2 V vs. LIG, acquisition time of 60 s and no further incubation time A slope of -0.32 ± 0.01 µA·L·mmol^{-1} and an adjusted R^2 value of 0.9895 were calculated. The LOD is 120 ± 4 µmol·L^{-1} and the LOQ is 365 ± 11 µmol·L^{-1}

Lactate Biosensors' Performance Under Different Conditions

The simple exchangeability of the enzyme was proven by replacing GOx with LOx. The lactate detection with LOx immobilized was performed within a concentration range of 10 µmol·L^{-1} and 5 mmol·L^{-1}. The resulting calibration curve for lactate detection with droplet method is shown in Fig. S22 a. A LOD of 28 ± 3 µmol·L^{-1} and a LOQ of 86 ± 8 µmol·L^{-1} were reached. The obtained sensitivity is 16 ± 1 µA·L·mmol^{-1}·cm^{-2}. Especially the lower quantification limit is completely satisfying for sensing in sweat.

Gauze was tested as sweat collecting material due to the unsatisfying results with filter paper as soaking material. It was necessary to use 60 µL of sample solution to wet the whole gauze pad. The results of a triple measurement on one sensor in comparison to droplet method are shown in Fig. S22 b.

A triple measurement of different lactate concentrations ranging between 0.1 and 5 mmol·L^{-1} in pure buffer and in a synthetic sweat matrix according to DIN 53160-2 was performed on one sensor, respectively. Droplet method was used for both investigations. The resulting dose-response curves are shown in Fig. S22 c. The curve shapes of both data sets are very similar. The curve of synthetic sweat spiked with lactate is shifted parallel to lower currents compared to the measurements of lactate in 1X PBS pH 7.4.

Lactate is also present in human tears. Therefore, lactate determination was performed in an artificial tear fluid. The used commercial eye drops contain 0.15% sodium hyaluronate and sodium chloride in a citrate buffer around pH 7. The pure solution was used as solvent for different lactate concentrations between 0.01 mmol·L^{-1}and 2.5 mmol·L^{-1}. The results in comparison to lactate in 1X PBS pH 7.4 are shown in Fig. S22 d. Both determinations were performed on the same sensor to enable a direct comparison of the curves. The curve shapes of both data sets are related and comparable.

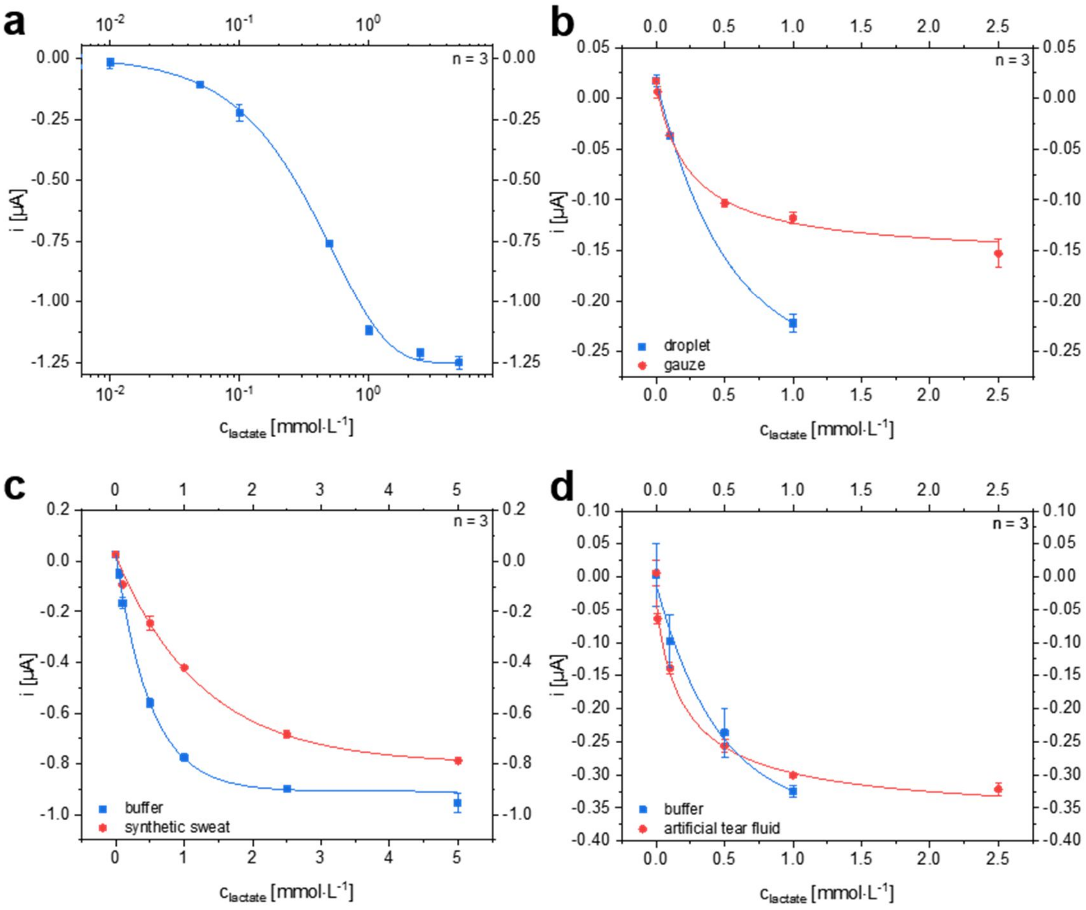

Fig. S22 a Dose-response curve (n = 3, SD represented by error bars, droplet method) for amperometric detection of lactate at a potential of -20 mV vs. LIG and an acquisition time of 60 s on half-logarithmic scale. The biosensor was modified with chemical deposited Prussian blue and a 0.1% chitosan membrane. LOx activity on the WE was 1.4 U·mm^{-2}. There was no further incubation time **b** Lactate determination in 1X PBS pH 7.4 with the droplet method (blue boxes) and gauze as sweat collecting pad (red circles). Chronoamperometry was performed at a potential of -20 mV vs. LIG. The tests were performed three times for each concentration (n = 3) on the same sensor to compare the results directly **c** Triplicate measurement of lactate in pure 1X PBS pH 7.4 (blue boxes) and dilutions in synthetic sweat solution pH 6.5 (red circles) according to DIN 53160-2 with the same lactate sensor at E = -50 mV vs. LIG **d** Lactate determination with the droplet method in 1X PBS pH 7.4 (blue boxes) and in artificial tear fluid (red circles). Tests were performed three times for each concentration on the same sensor to compare the results directly

Characterization of Electrode Material

Surface morphology and graphene-like characteristics of the LIG was determined by SEM imaging and Raman spectroscopy. The SEM images (Fig. S23 a, b and c) show the same spot of the LIG with different magnifications. One can clearly see the roughness and porosity of the three dimensional material. Furthermore, thin sheets and flakes are visible. For further characterization, Raman spectra were recorded (Fig. S23 d). The pronounced 2D peak at around 2700 cm^{-1} is comparable with the one in single-layer graphene. The 2D band profile can be typically found in 2D graphite with randomly stacked graphene layers along the c-axis [24].

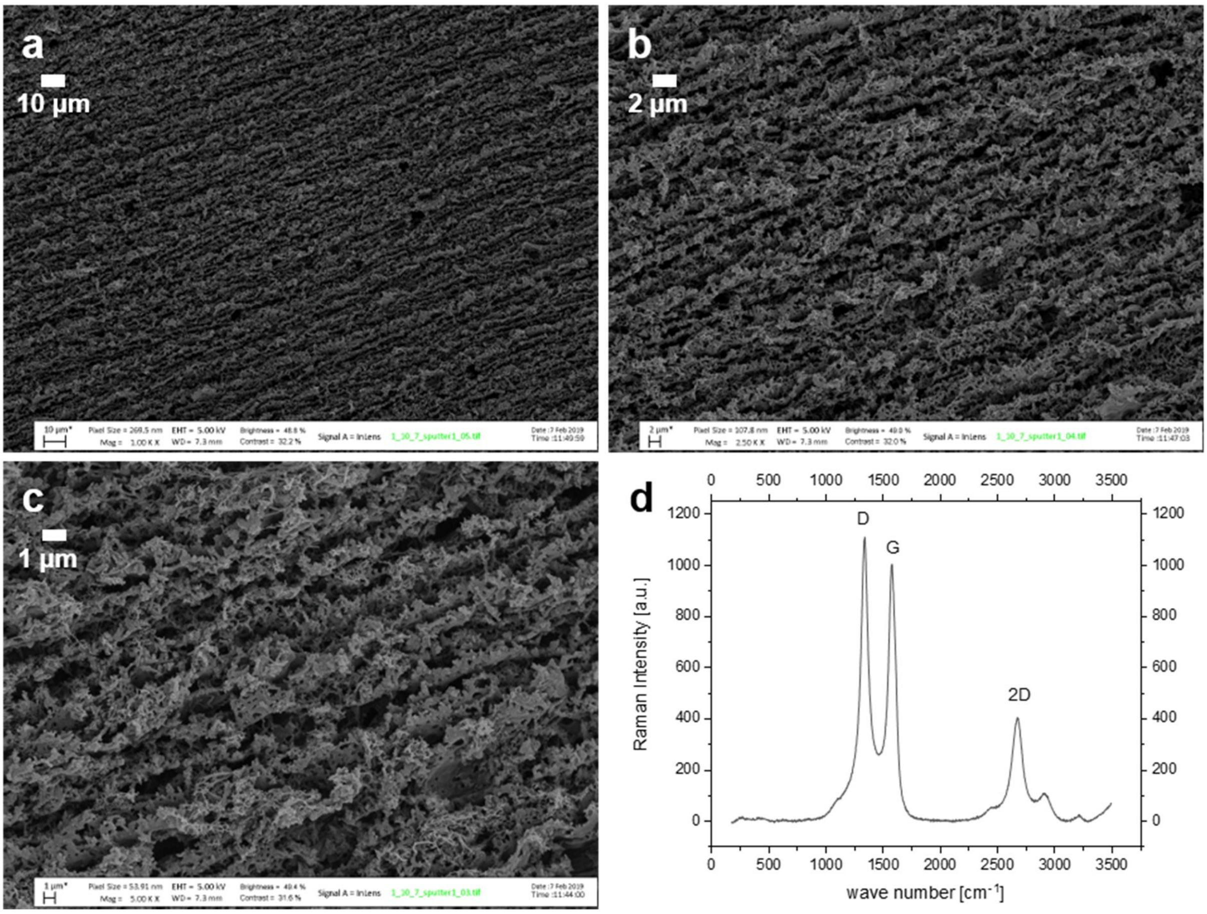

Fig. S23 SEM images (magnification factors **a** 1,000 **b** 2,500 **c** 5,000). The porous and rough structure of the LIG is clearly visible. **d** Raman spectra of the LIG structure (n = 16, error bars are hidden for clarity, 532 nm laser, set at 8 mW, 50 µm slit aperture, 50 X objective)

References

24. Lin J, Peng Z, Liu Y, Ruiz-Zepeda F, Ye R, Samuel ELG, Yacaman MJ, Yakobson BI, Tour JM. Laser-induced porous graphene films from commercial polymers. Nat. Commun. 2014:5:5714–5722.
29. Gao W, Emaminejad S. Fully integrated wearable sensor arrays for multiplexed in situ perspiration analysis. Nature. 2016:529:509–513.
33. Zhu J, Zhu Z, Lai Z, Wang R, Guo X, Wu X, Zhang G, Zhang Z, Wang Y, Chen Z. Planar Amperometric Glucose Sensor Based on Glucose Oxidase Immobilized by Chitosan Film on Prussian Blue Layer. Sensors. 2002:2(4):127–136.
34. Sempionatto JR, Nakagawa T, Pavinatto A, Mensah ST, Imani S, Mercier P, Wang J. Eyeglasses based wireless electrolyte and metabolite sensor platform. Lab. Chip. 2017:17(10):1834–1842.
35. Kulkarni T, Slaughter G. Application of Semipermeable Membranes in Glucose Biosensing. Membranes. 2016:6(4):55–75.
56. Timmer B, Sparreboom W, Olthuis W, Bergveld P, van den Berg A. Optimization of an electrolyte conductivity detector for measuring low ion concentrations. Lab Chip. 2020:2(2):121–124.
57. Ricci F, Moscone D, Palleschi G. Procedure 17 Preparation of Prussian blue-modified screen-printed electrodes via a chemical deposition for mass production of stable hydrogen peroxide sensors. Compr. Anal. Chem. 2007:49: e119-e124.
58. Jiang Y, Zhang X, Shan C, Hua S, Zhang Q, Bai X, Dan L, Niu L. Functionalization of graphene with electrodeposited Prussian blue towards amperometric sensing application. Talanta. 2011:85(1):76–81.
59. Jia W-Z, Wang K, Xia X-H. Elimination of electrochemical interferences in glucose biosensors. Trends Analyt. Chem. 2010:29(4):306–318.
60. Zhang X, Wang J, Ogorevc B, Spichiger UE. Glucose Nanosensor Based on Prussian-Blue Modified Carbon-Fiber Cone Nanoelectrode and an Integrated Reference Electrode. Electroanalysis. 1999:11(13):945–949.
